# Statistical (n,$\gamma$) cross section model comparison for short-lived nuclei

**DOI:** 10.1140/epja/s10050-023-00920-0

**Published:** 2023-03-09

**Authors:** R. Lewis, A. Couture, S. N. Liddick, A. Spyrou, D. L. Bleuel, L. Crespo Campo, B. P. Crider, A. C. Dombos, M. Guttormsen, T. Kawano, A. C. Larsen, A. M. Lewis, S. Mosby, G. Perdikakis, C. J. Prokop, S. J. Quinn, T. Renstrøm, S. Siem

**Affiliations:** 1 National Superconducting Cyclotron Laboratory, Michigan State University, East Lansing, MI 48824 USA; 2 Department of Chemistry, Michigan State University, East Lansing, MI 48824 USA; 3 Los Alamos National Laboratory, Los Alamos, NM 87545 USA; 4 Department of Physics and Astronomy, Michigan State University, East Lansing, MI 48824 USA; 5 Joint Institute for Nuclear Astrophysics, Michigan State University, East Lansing, MI 48824 USA; 6 Lawrence Livermore National Laboratory, Livermore, CA 94550 USA; 7 Department of Physics, University of Oslo, 0316 Oslo, Norway; 8 Department of Nuclear Engineering, University of California Berkeley, Berkeley, CA 94720 USA; 9 Central Michigan University, Mount Pleasant, MI 48859 USA; 10 Present Address: Zeno Power Systems, Inc., Washington, DC USA; 11 Present Address: Naval Nuclear Laboratory, New York, USA

## Abstract

Neutron-capture cross sections of neutron-rich nuclei are calculated using a Hauser–Feshbach model when direct experimental cross sections cannot be obtained. A number of codes to perform these calculations exist, and each makes different assumptions about the underlying nuclear physics. We investigated the systematic uncertainty associated with the choice of Hauser-Feshbach code used to calculate the neutron-capture cross section of a short-lived nucleus. The neutron-capture cross section for $^{73}\hbox {Zn}$(n,$\gamma$)$^{74}\hbox {Zn}$ was calculated using three Hauser-Feshbach statistical model codes: TALYS, CoH, and EMPIRE. The calculation was first performed without any changes to the default settings in each code. Then an experimentally obtained nuclear level density (NLD) and $\gamma$-ray strength function ($\gamma \hbox {SF}$) were included. Finally, the nuclear structure information was made consistent across the codes. The neutron-capture cross sections obtained from the three codes are in good agreement after including the experimentally obtained NLD and $\gamma \hbox {SF}$, accounting for differences in the underlying nuclear reaction models, and enforcing consistent approximations for unknown nuclear data. It is possible to use consistent inputs and nuclear physics to reduce the differences in the calculated neutron-capture cross section from different Hauser-Feshbach codes. However, ensuring the treatment of the input of experimental data and other nuclear physics are similar across multiple codes requires a careful investigation. For this reason, more complete documentation of the inputs and physics chosen is important.

**Supplementary Information:**

The online version contains supplementary material available at 10.1140/epja/s10050-023-00920-0.

## Introduction

The astrophysical slow and rapid neutron-capture processes (s- and r-processes, respectively) are responsible for the majority of the abundance of the elements heavier than iron [1–3]. Both processes proceed through unstable nuclei and require knowledge of neutron-capture cross sections [4] that are not available by direct measurement techniques. This leads to a reliance on theoretical cross section values for short-lived isotopes. Theoretical calculations of the neutron-capture cross section can vary for stable nuclei [5], and the variation only gets larger further from stability [4, 6–8]. Statistical neutron capture can be described using a Hauser-Feshbach model [9] that requires an optical model potential (OMP) describing the interaction between the neutron and nucleus, as well as two important statistical properties of the compound nucleus: the nuclear level density (NLD) and $\gamma$-ray strength function ($\gamma \hbox {SF}$). The NLD describes the number of levels per unit energy in a nucleus, and increases exponentially with excitation energy. The $\gamma \hbox {SF}$, which is related to the $\gamma$-ray transmission coefficient, describes the probability that a $\gamma$-ray of a given energy will be emitted from the nucleus. There are a number of models in use to describe the NLD and $\gamma \hbox {SF}$ of nuclei [10], each with parameters that can be varied, which leads to many different ways to describe the shape and magnitude of both the NLD and $\gamma \hbox {SF}$ for a given nucleus.

There are many Hauser-Feshbach statistical model codes available for calculating neutron-capture cross sections, including TALYS [10, 11], NON-SMOKER [12, 13], EMPIRE [14], CoH [15], SAPPHIRE [16], and CIGAR [5]. In each, a range of models for the NLD, $\gamma \hbox {SF}$, and OMP are provided. A comparison of the neutron-capture cross sections calculated using four Hauser-Feshbach codes (TALYS, NON-SMOKER, CIGAR, and SAPPHIRE) on stable nuclei demonstrated that, as expected, variations in model parameters between the different codes result in variations in the neutron-capture cross sections [5]. Differences of a factor of 2–3 at 30 keV were found for nuclei at or near stability.

Investigating the discrepancy between Hauser-Feshbach codes becomes more complicated further from stability due to the potential for large uncertainties in the extrapolation of NLD and $\gamma \hbox {SF}$ and the decreasing amount of experimentally known nuclear data. Even when nuclei are well studied, such as $^{238}\hbox {U}$ and $^{239}\hbox {Pu}$, it can be difficult to control for all possible differences between codes [17]. Despite the difficulties, the lack of directly measured neutron-capture cross sections off stability makes such an investigation important. Experimental efforts have focused on constraining the NLD and $\gamma \hbox {SF}$, as they contribute the largest uncertainty to cross sections obtained from Hauser-Feshbach codes [18–20]. This can reduce the uncertainty in neutron-capture cross sections dramatically, even far from stability [7], but that reduction has so far only been achieved inside the framework of a single Hauser-Feshbach code.

Together this presents a set of real challenges to address in order to proceed. One, there is a clear need for reliable neutron capture cross section predictions far from stability. Two, at present, there is no experimental capability for direct cross section measurements on these unstable isotopes comparable to what has been performed over the last 60 years for stable isotopes. Instead, rapid development of experimental techniques on unstable isotopes has offered new constraints on the model parameters used to calculate neutron capture cross sections. In contrast to a direct measurement, these measurements must necessarily be interpreted in the framework of a reaction model in order to comment on their impact on the unknown cross sections.

In this manuscript, we will discuss how the results from one such measurement can be used to understand differences between the predictions from different statistical model codes. We will illustrate how remaining nuclear data uncertainties give rise to remaining uncertainties in the cross section predictions. Finally, we will make recommendations on how experimental results from studies designed to inform Hauser-Feshbach model code predictions should report both results and calculation parameters in order to make it possible to compare predictions from different codes. We report on the first such comparison on neutron-rich nuclei, using the $^{73}\hbox {Zn}$(n,$\gamma$)$^{74}\hbox {Zn}$ reaction as a test case. While such comparisons are also possible on nuclei near stability, near stability there is general agreement on the underlying nuclear data in the calculations. $^{73}\hbox {Zn}$ is only three units from stability, however, the limitations in data are more representative of the situation for a nucleus on the *r*-process path.

## Comparison of Hauser-Feschbach statistical model codes

Three Hauser-Feshbach statistical codes were chosen to compare a calculated neutron-capture cross section: TALYS (v. 1.6), EMPIRE (v. 3.2.3), and CoH (v. 3.5.1). Adapting codes to use identical nuclear inputs in the same manner is not straightforward. In the previous study [5] the authors instead developed two new codes (CIGAR and SAPPHIRE) to guarantee identical treatment of nuclear inputs, and investigated the uncertainty in the Maxwellian-averaged cross section (MACS) resulting from differences such as how many of the known experimental levels were used in the calculation [5]. CoH, EMPIRE, and TALYS have a number of differences in physics input, model assumptions, and restrictions that were investigated.

**Fig. 1:**
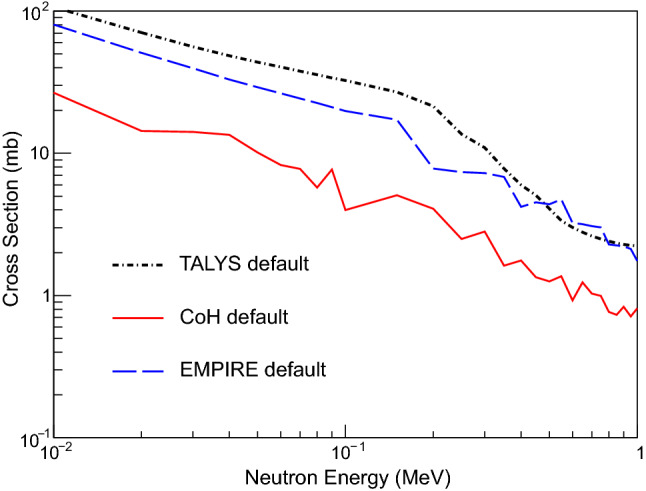
Comparison of $^{73}\hbox {Zn}$(n,$\gamma$)$^{74}\hbox {Zn}$ cross section calculated using TALYS, EMPIRE, and CoH without altering any of the default settings

**Fig. 2:**
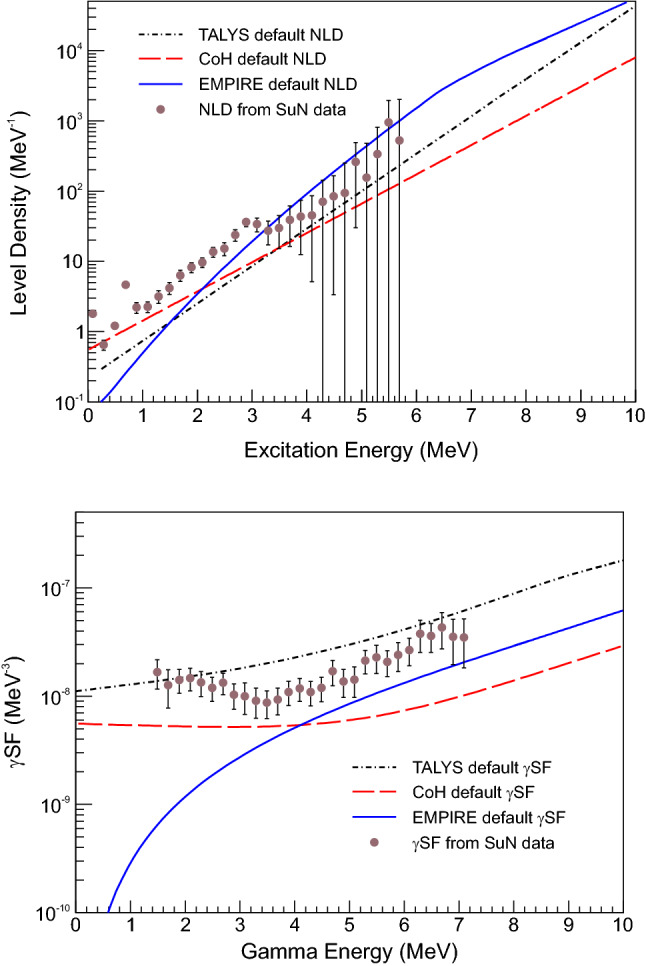
(Top) Default NLD for $^{74}\hbox {Zn}$ in TALYS, CoH, and EMPIRE compared to the experimental NLD obtained using the $\beta$-Oslo method [21]. (Bottom) Default $\gamma \hbox {SF}$ for $^{74}$Zn in TALYS, CoH, and EMPIRE compared to the experimental $\gamma$SF obtained using the $\beta$-Oslo method [21]

In the present study, the codes were first compared using a “black box” approach where as little information as possible was given to run the calculation. All of the default conditions, including the shape of the NLD and $\gamma \hbox {SF}$, were not changed. As shown in Fig. 1, the shape and magnitude of the results of the default calculations do not agree. This points to differences in both the models for the NLD and $\gamma \hbox {SF}$ (the defaults are very different between the codes, see Fig. 2), as well as some of the nuclear structure information that is being used in the calculations. For many unstable nuclei, this comparison gives a reasonable representation of the minimum level of uncertainty before a dedicated measurement to improve the statistical model parameters on a particular nucleus. For detailed tables of the NLD and $\gamma \hbox {SF}$ values, see the Supplemental Information.

### Incorporating experimental data

The experimentally constrained NLD and $\gamma$SF from Ref. [21] were used for this comparison. A $^{74}$Cu $\beta$-decay experiment was performed to extract the NLD and $\gamma$SF of $^{74}$Zn using the $\beta$-Oslo method [22]. The $\gamma$ rays observed in the Summing NaI detector (SuN) [23] provided the statistical decay information needed to determine the NLD and $\gamma$SF. This was the first experimental data for the NLD and $\gamma$SF of $^{74}$Zn, which allowed the shape of both to be better constrained in the Hauser-Feshbach statistical codes.

**Fig. 3:**
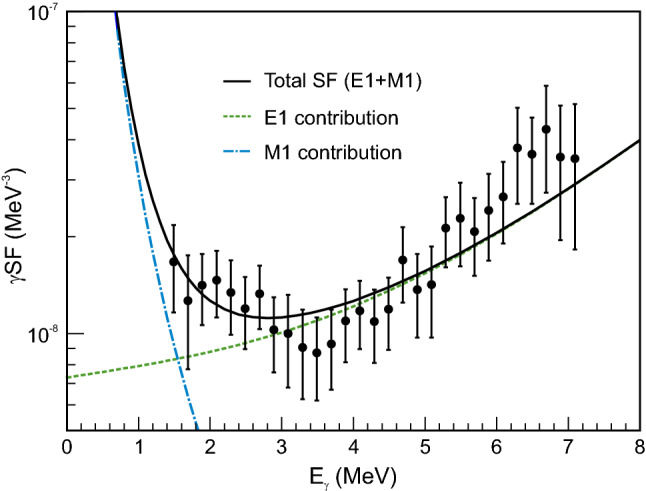
$\gamma$SF for $^{74}$Zn from Ref. [21]. The experimental data were fit with a GLO E1 and a SLO M1 (see text for details) and the parameters of the fit are shown in Table 1

The NLD, shown in Fig. 2, was formatted so that it could be read in to each code without any changes or renormalizations. TALYS has options for both spin-dependent and spin- and parity-dependent tables to read in the NLD, but because there was no information about the parity distribution from the experimental data, only a spin-dependent NLD was used, which assumed an equal parity distribution. CoH and EMPIRE required a spin- and parity-dependent table, which was generated with an equal parity distribution to match the NLD for TALYS. The spin dependence developed by Gilbert and Cameron [24] was used in all cases. The $\gamma$SF was restricted in CoH to a combination of a generalized Lorentzian (GLO) [25] function for the E1 component and a standard Lorentzian (SLO) [26] function for the M1 upbend component. The E1 parameters were restricted based on a GLO fit to photoabsorption cross section data [27], then the GLO+SLO function was fit to the $\gamma$SF data. The parameters from the fit (Fig. 3) are shown in Table 1. In TALYS the function needed to be added directly to the source code due to constraints on the M1 SLO parameter values, while in CoH the GLO and SLO parameters were defined in the input file. EMPIRE allows for the $\gamma$SF to be read in from a table, which was generated using the GLO+SLO function and fit parameters. The NLD and $\gamma$SF were checked at various stages in the calculation to make sure no further adjustments were made in the calculation process.

**Table 1:** GLO E1 parameters and SLO M1 parameters used to fit the experimental $\gamma$SF

Parameter	Value
$$\hbox {E}_{{E1}}$$ (MeV)	17.7(1)
$$\sigma _{E1}$$ (mb)	98(2)
$$\varGamma _{E1}$$ (MeV)	11.2(6)
$\hbox {E}_{{M1}}$ (MeV)	4.63 $\times$ 10$^{-7}$(500)
$$\sigma _{M1}$$ (mb)	39(9)
$\varGamma _{M1}$ (MeV)	0.096(88)

**Fig. 4:**
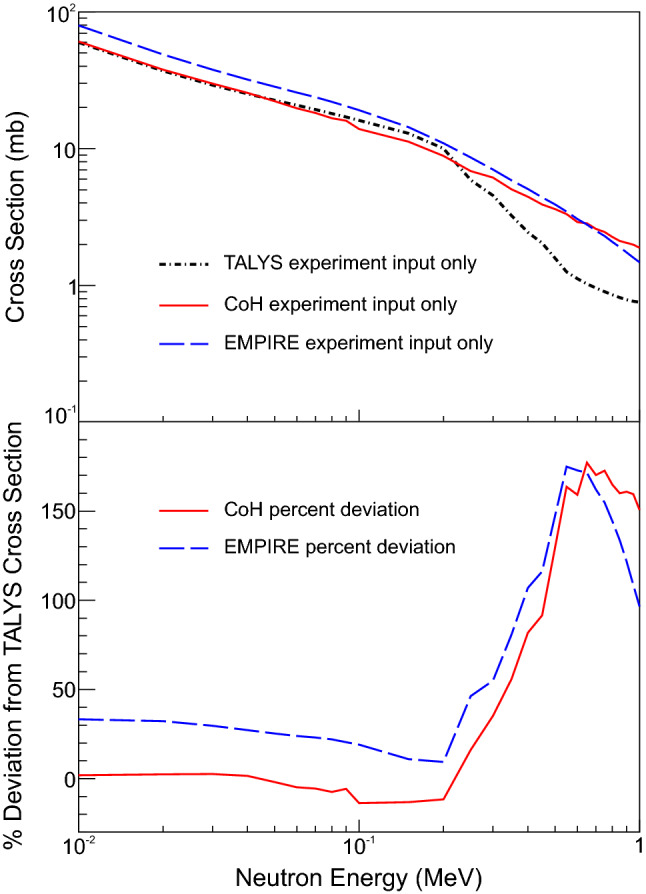
(Top) TALYS, EMPIRE, and CoH $^{73}$Zn(n,$\gamma$)$^{74}$Zn cross sections using experimentally obtained NLD and $\gamma$SF. All other aspects of the codes were left to default conditions. (Bottom) Percent deviation of the CoH and EMPIRE cross sections compared to the TALYS cross section after including the experimental NLD and $\gamma$SF

The cross sections from each code after adding in the experimentally-constrained NLD and $\gamma$SF are shown in Fig. 4. It is evident that much of the discrepancy in the cross sections between Fig. 1 and Fig. 4 came from the default choices for the parameters in the NLD and $\gamma$SF. With experimental data the cross sections are in much better agreement, especially at lower neutron energies (e.g., below 100 keV). This suggests that by requiring the three codes to use exactly the same NLD and $\gamma$SF, the large discrepancy between the calculated cross sections can be limited. However, there are still differences in the shape of the cross section at neutron energies above 100 keV. The bottom portion of Fig. 4 shows the percent deviation of the CoH and EMPIRE cross sections compared to the TALYS cross section. The deviation ranges from $-14\%$ to 175%, with the largest deviation occurring at higher neutron energies. A particularly large change can be seen around 195 keV, the energy of the first excited state in $^{73}$Zn, of which the spin and parity was only tentatively assigned as a (5/2$^+$) [28].

**Table 2:** $^{73}$Zn level energies, spins, and parities including the values assigned in TALYS, CoH, and EMPIRE, as well as the RIPL-3 suggested levels and the values used in the final comparison

$^{73}$Zn level energy (keV)	Suggested spin/parity (RIPL-3)	Final spin/parity used	TALYS spin/parity	CoH spin/parity	EMPIRE spin/parity
0	(1/2$^-$)	1/2$^-$	1/2$^-$	1/2$^-$	1/2$^-$
195.5$^{ab}$	(5/2$^+$)	5/2$^+$	5/2$^+$	3/2$^+$	No assignment
307.2$^{ab}$	(5/2)	5/2$^+$	5/2$^+$	No assignment	No assignment
337$^{ab}$	None	3/2$^+$	3/2$^+$	3/2$^-$	No assignment
449.6$^{ab}$	(3/2$^-$)	3/2$^-$	3/2$^-$	No assignment	No assignment
502.2$^{ab}$	(5/2)	5/2$^-$	5/2$^-$	No assignment	No assignment
1124$^{ab}$	(5/2)	5/2$^-$	5/2$^-$	No assignment	No assignment
2008.9$^{ab}$	(5/2)	5/2$^-$	5/2$^-$	No assignment	No assignment

$^\textrm{a}$Level not used by CoH

$^\textrm{b}$Level not used by EMPIRE

**Table 3:** $^{74}$Zn level energies, spins, and parities including the values assigned in TALYS, CoH, and EMPIRE, as well as the RIPL-3 suggested levels and the values used in the final comparison

$^{74}$Zn level energy (keV)	Suggested spin/parity (RIPL-3)	Final spin/parity used	TALYS spin/parity	CoH spin/parity	EMPIRE spin/parity
0	0$^+$	0$^+$	0$^+$	0$^+$	0$^+$
605.9	2$^+$	2$^+$	2$^+$	2$^+$	2$^+$
1418.56$^{a}$	(0$^+$, 4$^+$)	4$^+$	3$^+$	No assignment	0$^+$
1670.25	(2$^+$)	2$^+$	2$^+$	2$^+$	2$^+$
1788.9$^{a}$	None	1$^+$	1$^+$	No assignment	0$^+$
2099.23$^{a}$	None	4$^-$	4$^-$	No assignment	0$^+$
2148.2$^{a}$	(1, 2$^+$)	2$^+$	5$^-$	No assignment	0$^+$
2353.6$^{a}$	None	3$^+$	3$^+$	No assignment	0$^+$
2551.88$^{ab}$	None	1$^+$	1$^+$	No assignment	No assignment
2657.6$^{ab}$	None	6$^-$	6$^-$	No assignment	No assignment
2698$^{ab}$	None	1$^+$	4$^-$	No assignment	No assignment
2809.04$^{ab}$	None	2$^-$	2$^-$	No assignment	No assignment
2904.73$^{ab}$	None	3$^-$	3$^-$	No assignment	No assignment
2969.3$^{ab}$	None	1$^-$	1$^-$	No assignment	No assignment
2985.9$^{ab}$	None	7$^+$	7$^+$	No assignment	No assignment
3063.9$^{ab}$	None	2$^-$	2$^-$	No assignment	No assignment
3067$^{ab}$	None	5$^+$	5$^+$	No assignment	No assignment
3571$^{ab}$	None	3$^-$	3$^-$	No assignment	No assignment
4562.4$^{ab}$	None	4$^+$	4$^+$	No assignment	No assignment
4861.8$^{ab}$	None	1$^-$	1$^-$	No assignment	No assignment
4896.8$^{ab}$	None	2$^+$	2$^+$	No assignment	No assignment
5628$^{ab}$	None	0$^-$	0$^-$	No assignment	No assignment

$^\textrm{a}$ Level not used by CoH

$^\textrm{b}$ Level not used by EMPIRE

### Spin and parity assignments

The spins and parities of levels in the nuclei involved had an impact on the cross sections. In both $^{73,74}$Zn many of the levels included in the RIPL-3 database [29] have unknown spins and parities. Tables 2 and 3 list the known excited states of both nuclei, along with the spins and parities suggested in RIPL-3, the final spins and parities used in the comparison calculation, and the original assignments made by each of the three codes. The first excited state of $^{73}$Zn at 195.5 keV for example, shows how large the impact of unknown spins and parities can be. Both CoH and EMPIRE did not consider the 195.5 keV level for inelastic neutron scattering due to the lack of a firm $\hbox {J}^{\pi }$ assignment, while TALYS used the level with its tentative assignment for inelastic neutron scattering. The CoH and EMPIRE cross sections deviate from the TALYS cross section significantly at this energy. By giving the level a 5/2$^+$ assignment, which is what TALYS had already assigned the level, inelastic scattering to the first excited state in $^{73}$Zn became possible in the calculated cross sections from EMPIRE and CoH. The 5/2$^+$ assignment has been recently confirmed [30, 31]. The updated $\hbox {J}^{\pi }$ value for the level described highlights the importance of continually updating cross sections as nuclear data improves, as it can impact statistical calculations.

TALYS assigns a spin and parity based on statistical spin rules, while CoH uses statistical spin rules to make an assignment up to a certain number of levels, which is specified in a level file for each nucleus. EMPIRE also assigns a spin and parity up to a certain number of levels, though for the even-even nucleus $^{74}$Zn all of the assignments were 0$^{+}$. Assignments do not always match those suggested in RIPL-3 and are not consistent between the three codes. As an additional example, RIPL-3 suggests a spin and parity assignment of (0$^+$, 4$^+$) for the second excited state at 1418 keV in $^{74}$Zn, but the assignment in TALYS was 3$^+$, EMPIRE made an assignment of 0$^{+}$, and CoH made no assignment at all. For the purpose of the present comparison, levels that had unknown spins/parities were assigned a value that was the same across all three codes. The 1418 keV level was assigned a spin and parity of 4$^+$ due to an updated ENSDF evaluation in 2017 that suggested a 4$^+$ assignment from Coulomb excitation [32], though the spin change had no impact on the cross section calculation.

It is generally assumed that not all the excited states of a nucleus have been observed, especially for nuclei far from stability. TALYS uses the discrete levels provided in the RIPL-3 table, then creates levels at higher energies until there are at least 100 discrete levels. The levels are created based on the microscopic level density of Goriely, Hilaire, and Koning calculated using the Hartree-Fock-Bogoliubov plus combinatorial method using the Skyrme force [33]. The addition of the theoretical levels is a default setting, but can be turned off in the input file. CoH provides the option to populate the nucleus with a continuum of states above a given level, which can start at a lower energy than the highest level in RIPL-3. The continuum is based on the level density that is being used in the calculation. This is not an option that can be changed in the input file, but is still easily modified in the list of levels that is read in. Finally, EMPIRE does not provide any discrete levels beyond those in RIPL-3. For the present comparison, only the levels in RIPL-3 were included, with spins and parities of those levels edited to be consistent among the codes. While new structure information is always becoming available (e.g., for $^{73}\hbox {Zn}$), there are still many neutron-rich nuclei that need to be studied so that there is complete low- and high-energy structure information that is vital for cross section calculations.

## Discussion

**Fig. 5:**
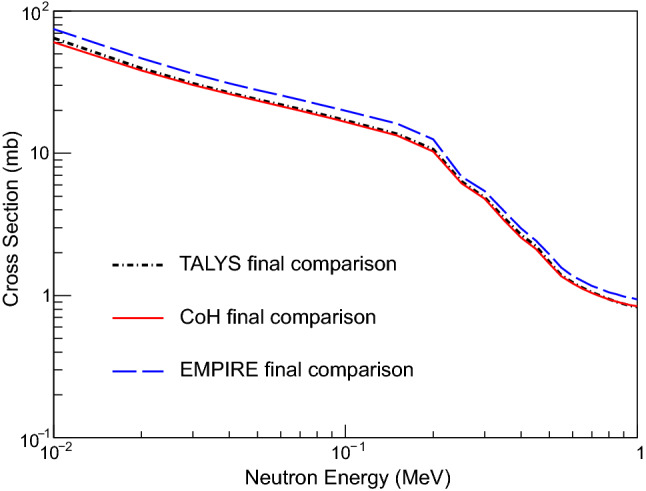
Final cross section comparison of TALYS, EMPIRE, and CoH using experimentally obtained NLD and $\gamma$SF. Other parameters were also changed to be consistent between the three codes, including spins and parities of levels (see text for detail)

After accounting for all of the differences, the calculated neutron-capture cross sections from TALYS, CoH, and EMPIRE agree well, as shown in Fig. 5. The small discrepancy in the EMPIRE cross section, which is about 10% higher than those of CoH and TALYS, is most likely due to the different width fluctuation corrections (WFC) that are used in the calculations. CoH uses WFC described in [34], which are based on the Moldauer approach that is used in TALYS [35], which leads to very similar treatments and therefore very similar cross sections. EMPIRE uses the Hofmann, Richert, Tepel, and Weidenmüller (HRTW) approach [36, 37], which was shown to differ from the Moldauer formulation [34, 38].

Producing a neutron-capture cross section that incorporates experimental data for the NLD and $\gamma$SF requires choosing a code that allows for the data to be included as accurately as possible; the choice also brings in a set of assumptions such as those detailed in Table 2. A casual user is more likely to arrive at disagreements resembling Fig. 1 than to reach results similar to those in Fig. 5. A dedicated experiment to improve the uncertainties in predictions for unstable nuclei would likely achieve the level of agreement observed in Fig. 4. However, if only one code is used, the systematic uncertainty explored here is still unknown.

This points to a dilemma in reaction modeling for cases where the underlying nuclear data (such as level energies, spins, and parities) are not well known. One path is to force the inclusion of known physical processes, even when the data are inadequate to calculate them properly. Another option is to only calculate those processes where the data are adequate for a robust calculation. To be clear, this is not a deficiency in the model, but rather a deficiency in the nuclear data used in the calculation. Both choices are reasonable assumptions under adverse conditions; the choice is likely driven by the primary physics problems being addressed. These two options are illustrated in the differing treatments for inelastic scattering in $^{73}$Zn where spectroscopic level information is incomplete. Improving agreement beyond what is observed in Fig. 4 requires improved nuclear data.

Based on these observations, we recommend that experimental work using statistical model codes to interpret the measurements report not only the quantities measured in the experiment, but the additional nuclear data used in the statistical model calculations. In the case of $^{73}$Zn discussed here, that would mean reporting the $\gamma$SF parameters from Table 1 as well as the structure parameters from Tables 2 and 3 taken from literature and used in the interpretation. In this way, later work can make direct comparisons and clearly differentiate differences arising from input data versus those arising from code techniques. Finally, this gives a clearer picture of where additional measurements could improve the uncertainties in theoretical predictions.

## Conclusions

As demonstrated, it is possible to obtain the same cross section (within 10%) with TALYS, CoH, and EMPIRE for a neutron-rich nucleus using experimentally obtained inputs. This ultimately indicates that the choice of Hauser-Feshbach code has a small impact on the overall uncertainty of the calculated cross section, as expected, when the differing assumptions are addressed. However, there are inherent challenges in calculating neutron-capture cross sections in regions where the underlying nuclear data are incomplete. A calculation must necessarily make assumptions about unknown nuclear quantities in order to complete the calculation. In the case of $^{73}$Zn(n, $\gamma$)$^{74}$Zn, even after identical experimentally derived NLD and $\gamma$SF were incorporated into TALYS, CoH, and EMPIRE, the calculated results differed in scale and energy dependence. This was due to the underlying assumptions about the existence and properties of states in the initial and final nucleus, which were all supported by a robust physics justification. Further, each code has different restrictions on how to incorporate the experimental data. The major cause of the deviation between codes after incorporating the experimental NLD and $\gamma$SF comes from the lack of information about low-lying levels, which is information that can be obtained for many neutron-rich nuclei with spectroscopic experiments. Concerns about accurately representing and incorporating data into the calculation can limit which codes can be used for a particular nucleus, and that choice brings with it the underlying assumptions of the code. Future measurements should provide more complete documentation of the nuclear data inputs chosen in the reaction model calculations when interpreting the experimental results.

## Supplementary Information

Below is the link to the electronic supplementary material.

Supplementary file 1 (pdf 109 KB)

## Data Availability

This manuscript has associated data in a data repository. [Authors’ comment: Tables of the default code NLD and $\gamma$SF for $^{73}$ZN are available in the supplementary material.]
