# Supplementary material for: Statistical (n,γ) cross section model comparison for short-lived nuclei
Source: Eur Phys J A Hadron Nucl. 2023 Mar 9;59(3):42. doi: 10.1140/epja/s10050-023-00920-0 (PMC9998597; doi:10.1140/epja/s10050-023-00920-0)
Supplement: Supplementary file 1 — (pdf 109 KB) [file 10050_2023_920_MOESM1_ESM.pdf]

Default NLD and γSF tables

Table 1: TALYS default NLD for ^{74}Zn including spin-dependent NLD for J=0-4. An equal parity distribution is assumed.

E_x (MeV)	Total NLD (MeV^{-1})	J=0	J=1	J=2	J=3	J=4
0.25	2.93E-01	2.15E-02	5.55E-02	6.86E-02	6.14E-02	4.34E-02
0.50	3.98E-01	2.82E-02	7.33E-02	9.15E-02	8.31E-02	6.00E-02
0.75	5.41E-01	3.71E-02	9.69E-02	1.22E-01	1.13E-01	8.27E-02
1.00	7.36E-01	4.89E-02	1.28E-01	1.63E-01	1.52E-01	1.14E-01
1.25	1.00E+00	6.45E-02	1.70E-01	2.18E-01	2.06E-01	1.57E-01
1.50	1.36E+00	8.50E-02	2.25E-01	2.90E-01	2.78E-01	2.15E-01
1.75	1.85E+00	1.12E-01	2.98E-01	3.88E-01	3.75E-01	2.94E-01
2.00	2.51E+00	1.48E-01	3.95E-01	5.18E-01	5.06E-01	4.02E-01
2.25	3.41E+00	1.96E-01	5.24E-01	6.91E-01	6.82E-01	5.50E-01
2.50	4.64E+00	2.60E-01	6.95E-01	9.24E-01	9.20E-01	7.51E-01
2.75	6.30E+00	3.44E-01	9.24E-01	1.23E+00	1.24E+00	1.02E+00
3.00	8.57E+00	4.56E-01	1.23E+00	1.65E+00	1.67E+00	1.40E+00
3.25	1.17E+01	6.04E-01	1.63E+00	2.20E+00	2.25E+00	1.90E+00
3.50	1.58E+01	8.02E-01	2.17E+00	2.95E+00	3.03E+00	2.59E+00
3.75	2.15E+01	1.07E+00	2.89E+00	3.94E+00	4.08E+00	3.52E+00
4.00	2.92E+01	1.41E+00	3.85E+00	5.27E+00	5.50E+00	4.78E+00
4.25	3.97E+01	1.88E+00	5.12E+00	7.05E+00	7.41E+00	6.49E+00
4.50	5.40E+01	2.50E+00	6.83E+00	9.44E+00	9.98E+00	8.82E+00
4.75	7.34E+01	3.32E+00	9.10E+00	1.26E+01	1.34E+01	1.20E+01
5.00	9.98E+01	4.42E+00	1.21E+01	1.69E+01	1.81E+01	1.62E+01
5.50	1.84E+02	7.85E+00	2.16E+01	3.03E+01	3.28E+01	2.99E+01
6.00	3.41E+02	1.39E+01	3.85E+01	5.44E+01	5.94E+01	5.49E+01
6.50	6.29E+02	2.48E+01	6.87E+01	9.76E+01	1.08E+02	1.01E+02
7.00	1.16E+03	4.42E+01	1.23E+02	1.75E+02	1.95E+02	1.85E+02
7.50	2.15E+03	7.88E+01	2.19E+02	3.15E+02	3.54E+02	3.38E+02
8.00	3.91E+03	1.37E+02	3.82E+02	5.53E+02	6.27E+02	6.08E+02
8.50	6.95E+03	2.33E+02	6.53E+02	9.51E+02	1.09E+03	1.07E+03
9.00	1.21E+04	3.89E+02	1.09E+03	1.60E+03	1.85E+03	1.83E+03
9.50	2.06E+04	6.40E+02	1.80E+03	2.65E+03	3.08E+03	3.08E+03
10.0	3.46E+04	1.04E+03	2.93E+03	4.32E+03	5.05E+03	5.10E+03
11.0	9.25E+04	2.61E+03	7.39E+03	1.10E+04	1.30E+04	1.33E+04
12.0	2.34E+05	6.26E+03	1.78E+04	2.66E+04	3.17E+04	3.29E+04
13.0	5.67E+05	1.44E+04	4.11E+04	6.19E+04	7.43E+04	7.78E+04
14.0	1.32E+06	3.21E+04	9.16E+04	1.39E+05	1.67E+05	1.77E+05
15.0	2.95E+06	6.91E+04	1.98E+05	3.00E+05	3.65E+05	3.89E+05
16.0	6.42E+06	1.45E+05	4.15E+05	6.32E+05	7.72E+05	8.28E+05
17.0	1.36E+07	2.96E+05	8.49E+05	1.30E+06	1.59E+06	1.72E+06

Continued on next page

Table 1 – continued from previous page

E_x (MeV)	Total NLD (MeV^{-1})	J=0	J=1	J=2	J=3	J=4
18.0	2.80E+07	5.91E+05	1.70E+06	2.60E+06	3.21E+06	3.48E+06
19.0	5.64E+07	1.16E+06	3.33E+06	5.11E+06	6.32E+06	6.89E+06
20.0	1.11E+08	2.22E+06	6.40E+06	9.85E+06	1.22E+07	1.34E+07
22.5	5.65E+08	1.06E+07	3.05E+07	4.72E+07	5.90E+07	6.53E+07
25.0	2.60E+09	4.61E+07	1.33E+08	2.07E+08	2.61E+08	2.91E+08
30.0	4.42E+10	7.11E+08	2.07E+09	3.23E+09	4.10E+09	4.63E+09
40.0	6.69E+12	9.31E+10	2.72E+11	4.28E+11	5.51E+11	6.34E+11
50.0	5.66E+14	7.03E+12	2.06E+13	3.26E+13	4.24E+13	4.93E+13
60.0	3.19E+16	3.61E+14	1.06E+15	1.69E+15	2.21E+15	2.59E+15
70.0	1.32E+18	1.39E+16	4.07E+16	6.50E+16	8.54E+16	1.01E+17
80.0	4.28E+19	4.20E+17	1.24E+18	1.98E+18	2.61E+18	3.11E+18
90.0	1.14E+21	1.05E+19	3.10E+19	4.97E+19	6.58E+19	7.86E+19
100	2.55E+22	2.24E+20	6.59E+20	1.06E+21	1.41E+21	1.69E+21
110	4.94E+23	4.13E+21	1.22E+22	1.96E+22	2.62E+22	3.14E+22
120	8.45E+24	6.76E+22	2.00E+23	3.22E+23	4.30E+23	5.18E+23
130	1.29E+26	9.93E+23	2.94E+24	4.74E+24	6.34E+24	7.66E+24
140	1.79E+27	1.33E+25	3.92E+25	6.34E+25	8.49E+25	1.03E+26
150	2.27E+28	1.63E+26	4.81E+26	7.78E+26	1.04E+27	1.27E+27
160	2.66E+29	1.84E+27	5.45E+27	8.83E+27	1.19E+28	1.44E+28
170	2.89E+30	1.94E+28	5.75E+28	9.33E+28	1.26E+29	1.53E+29
180	2.94E+31	1.92E+29	5.69E+29	9.24E+29	1.24E+30	1.52E+30
190	2.82E+32	1.79E+30	5.30E+30	8.62E+30	1.16E+31	1.42E+31
200	2.55E+33	1.58E+31	4.68E+31	7.60E+31	1.03E+32	1.25E+32

Table 2: TALYS default NLD for ^{74}Zn including spin-dependent NLD for J=5-8. An equal parity distribution is assumed.

E_x (MeV)	Total NLD (MeV^{-1})	J=5	J=6	J=7	J=8
0.25	2.93E-01	2.51E-02	1.21E-02	4.92E-03	1.69E-03
0.50	3.98E-01	3.56E-02	1.77E-02	7.44E-03	2.66E-03
0.75	5.41E-01	5.03E-02	2.57E-02	1.12E-02	4.14E-03
1.00	7.36E-01	7.08E-02	3.72E-02	1.67E-02	6.40E-03
1.25	1.00E+00	9.93E-02	5.35E-02	2.47E-02	9.80E-03
1.50	1.36E+00	1.39E-01	7.66E-02	3.63E-02	1.49E-02
1.75	1.85E+00	1.94E-01	1.09E-01	5.31E-02	2.24E-02
2.00	2.51E+00	2.70E-01	1.55E-01	7.74E-02	3.36E-02
2.25	3.41E+00	3.75E-01	2.20E-01	1.12E-01	5.00E-02
2.50	4.64E+00	5.20E-01	3.11E-01	1.62E-01	7.40E-02
2.75	6.30E+00	7.20E-01	4.38E-01	2.33E-01	1.09E-01
3.00	8.57E+00	9.95E-01	6.16E-01	3.34E-01	1.60E-01
3.25	1.17E+01	1.37E+00	8.63E-01	4.77E-01	2.33E-01
3.50	1.58E+01	1.89E+00	1.21E+00	6.79E-01	3.39E-01
3.75	2.15E+01	2.60E+00	1.69E+00	9.65E-01	4.91E-01
4.00	2.92E+01	3.58E+00	2.35E+00	1.37E+00	7.08E-01
4.25	3.97E+01	4.92E+00	3.27E+00	1.93E+00	1.02E+00
4.50	5.40E+01	6.75E+00	4.55E+00	2.73E+00	1.46E+00
4.75	7.34E+01	9.25E+00	6.31E+00	3.84E+00	2.09E+00
5.00	9.98E+01	1.27E+01	8.75E+00	5.39E+00	2.98E+00
5.50	1.84E+02	2.37E+01	1.67E+01	1.06E+01	6.02E+00
6.00	3.41E+02	4.43E+01	3.19E+01	2.06E+01	1.21E+01
6.50	6.29E+02	8.26E+01	6.06E+01	4.00E+01	2.40E+01
7.00	1.16E+03	1.54E+02	1.15E+02	7.72E+01	4.74E+01
7.50	2.15E+03	2.85E+02	2.16E+02	1.49E+02	9.31E+01
8.00	3.91E+03	5.22E+02	4.04E+02	2.85E+02	1.83E+02
8.50	6.95E+03	9.30E+02	7.33E+02	5.27E+02	3.48E+02
9.00	1.21E+04	1.62E+03	1.30E+03	9.49E+02	6.40E+02
9.50	2.06E+04	2.76E+03	2.24E+03	1.67E+03	1.15E+03
10.0	3.46E+04	4.60E+03	3.79E+03	2.86E+03	2.00E+03
11.0	9.25E+04	1.22E+04	1.03E+04	7.98E+03	5.74E+03
12.0	2.34E+05	3.07E+04	2.63E+04	2.08E+04	1.54E+04
13.0	5.67E+05	7.36E+04	6.40E+04	5.16E+04	3.88E+04
14.0	1.32E+06	1.69E+05	1.49E+05	1.22E+05	9.34E+04
15.0	2.95E+06	3.75E+05	3.34E+05	2.78E+05	2.16E+05
16.0	6.42E+06	8.07E+05	7.26E+05	6.10E+05	4.81E+05
17.0	1.36E+07	1.69E+06	1.53E+06	1.30E+06	1.04E+06
18.0	2.80E+07	3.44E+06	3.15E+06	2.70E+06	2.18E+06
19.0	5.64E+07	6.85E+06	6.33E+06	5.47E+06	4.46E+06
20.0	1.11E+08	1.34E+07	1.24E+07	1.08E+07	8.92E+06
22.5	5.65E+08	6.61E+07	6.24E+07	5.53E+07	4.64E+07
25.0	2.60E+09	2.97E+08	2.84E+08	2.55E+08	2.18E+08
30.0	4.42E+10	4.82E+09	4.69E+09	4.31E+09	3.77E+09
40.0	6.69E+12	6.74E+11	6.73E+11	6.39E+11	5.79E+11
50.0	5.66E+14	5.32E+13	5.42E+13	5.25E+13	4.87E+13
60.0	3.19E+16	2.83E+15	2.91E+15	2.87E+15	2.71E+15
70.0	1.32E+18	1.11E+17	1.16E+17	1.15E+17	1.10E+17
80.0	4.28E+19	3.44E+18	3.61E+18	3.63E+18	3.52E+18
90.0	1.14E+21	8.76E+19	9.26E+19	9.38E+19	9.17E+19
100	2.55E+22	1.89E+21	2.01E+21	2.05E+21	2.02E+21
110	4.94E+23	3.53E+22	3.78E+22	3.88E+22	3.84E+22

Continued on next page

Table 2 – continued from previous page

E_x (MeV)	Total NLD (MeV^{-1})	J=5	J=6	J=7	J=8
120	8.45E+24	5.85E+23	6.28E+23	6.47E+23	6.45E+23
130	1.29E+26	8.67E+24	9.34E+24	9.68E+24	9.70E+24
140	1.79E+27	1.17E+26	1.26E+26	1.31E+26	1.32E+26
150	2.27E+28	1.44E+27	1.56E+27	1.63E+27	1.65E+27
160	2.66E+29	1.64E+28	1.79E+28	1.87E+28	1.90E+28
170	2.89E+30	1.75E+29	1.90E+29	2.00E+29	2.03E+29
180	2.94E+31	1.74E+30	1.90E+30	2.00E+30	2.04E+30
190	2.82E+32	1.63E+31	1.78E+31	1.88E+31	1.92E+31
200	2.55E+33	1.44E+32	1.58E+32	1.67E+32	1.72E+32

Table 3: CoH default NLD for ^{74}Zn including spin-dependent NLD for J=0-3. An equal parity distribution is assumed.

E_x (MeV)	Total NLD (MeV^{-1})	J=0	J=1	J=2	J=3
2.46E-02	5.61E-01	5.25E-02	1.40E-01	1.85E-01	1.83E-01
4.46E-02	5.72E-01	5.35E-02	1.43E-01	1.89E-01	1.87E-01
6.46E-02	5.83E-01	5.46E-02	1.46E-01	1.93E-01	1.90E-01
8.46E-02	5.95E-01	5.56E-02	1.49E-01	1.96E-01	1.94E-01
1.05E-01	6.06E-01	5.67E-02	1.51E-01	2.00E-01	1.98E-01
1.25E-01	6.18E-01	5.78E-02	1.54E-01	2.04E-01	2.02E-01
1.45E-01	6.30E-01	5.89E-02	1.57E-01	2.08E-01	2.06E-01
1.65E-01	6.42E-01	6.00E-02	1.60E-01	2.12E-01	2.10E-01
1.85E-01	6.54E-01	6.12E-02	1.64E-01	2.16E-01	2.14E-01
2.05E-01	6.67E-01	6.24E-02	1.67E-01	2.20E-01	2.18E-01
2.25E-01	6.80E-01	6.36E-02	1.70E-01	2.25E-01	2.22E-01
2.45E-01	6.93E-01	6.48E-02	1.73E-01	2.29E-01	2.26E-01
2.65E-01	7.07E-01	6.61E-02	1.77E-01	2.33E-01	2.31E-01
2.85E-01	7.20E-01	6.74E-02	1.80E-01	2.38E-01	2.35E-01
3.05E-01	7.34E-01	6.87E-02	1.83E-01	2.42E-01	2.40E-01
3.25E-01	7.48E-01	7.00E-02	1.87E-01	2.47E-01	2.44E-01
3.45E-01	7.63E-01	7.13E-02	1.91E-01	2.52E-01	2.49E-01
3.65E-01	7.78E-01	7.27E-02	1.94E-01	2.57E-01	2.54E-01
3.85E-01	7.93E-01	7.41E-02	1.98E-01	2.62E-01	2.59E-01
4.05E-01	8.08E-01	7.56E-02	2.02E-01	2.67E-01	2.64E-01
4.25E-01	8.24E-01	7.70E-02	2.06E-01	2.72E-01	2.69E-01
4.45E-01	8.40E-01	7.85E-02	2.10E-01	2.77E-01	2.74E-01
4.65E-01	8.56E-01	8.00E-02	2.14E-01	2.83E-01	2.79E-01
4.85E-01	8.73E-01	8.16E-02	2.18E-01	2.88E-01	2.85E-01
5.05E-01	8.89E-01	8.32E-02	2.22E-01	2.94E-01	2.90E-01
5.25E-01	9.07E-01	8.48E-02	2.26E-01	2.99E-01	2.96E-01
5.45E-01	9.24E-01	8.64E-02	2.31E-01	3.05E-01	3.02E-01
5.65E-01	9.42E-01	8.81E-02	2.35E-01	3.11E-01	3.08E-01
5.85E-01	9.60E-01	8.98E-02	2.40E-01	3.17E-01	3.14E-01
6.05E-01	9.79E-01	9.15E-02	2.45E-01	3.23E-01	3.20E-01
6.25E-01	9.98E-01	9.33E-02	2.49E-01	3.29E-01	3.26E-01
6.45E-01	1.02E+00	9.51E-02	2.54E-01	3.36E-01	3.32E-01
6.65E-01	1.04E+00	9.70E-02	2.59E-01	3.42E-01	3.38E-01
6.85E-01	1.06E+00	9.88E-02	2.64E-01	3.49E-01	3.45E-01
7.05E-01	1.08E+00	1.01E-01	2.69E-01	3.56E-01	3.52E-01
7.25E-01	1.10E+00	1.03E-01	2.74E-01	3.63E-01	3.59E-01
7.45E-01	1.12E+00	1.05E-01	2.80E-01	3.70E-01	3.65E-01

Continued on next page

Table 3 – continued from previous page

E_x (MeV)	Total NLD (MeV^{-1})	J=0	J=1	J=2	J=3
7.65E-01	1.14E+00	1.07E-01	2.85E-01	3.77E-01	3.73E-01
7.85E-01	1.16E+00	1.09E-01	2.91E-01	3.84E-01	3.80E-01
8.05E-01	1.19E+00	1.11E-01	2.96E-01	3.92E-01	3.87E-01
8.25E-01	1.21E+00	1.13E-01	3.02E-01	3.99E-01	3.95E-01
8.45E-01	1.23E+00	1.15E-01	3.08E-01	4.07E-01	4.02E-01
8.65E-01	1.26E+00	1.17E-01	3.14E-01	4.15E-01	4.10E-01
8.85E-01	1.28E+00	1.20E-01	3.20E-01	4.23E-01	4.18E-01
9.05E-01	1.31E+00	1.22E-01	3.26E-01	4.31E-01	4.26E-01
9.25E-01	1.33E+00	1.24E-01	3.32E-01	4.39E-01	4.34E-01
9.45E-01	1.36E+00	1.27E-01	3.39E-01	4.48E-01	4.43E-01
9.65E-01	1.38E+00	1.29E-01	3.45E-01	4.56E-01	4.51E-01
9.85E-01	1.41E+00	1.32E-01	3.52E-01	4.65E-01	4.60E-01
1.01E+00	1.44E+00	1.34E-01	3.59E-01	4.74E-01	4.69E-01
1.03E+00	1.46E+00	1.37E-01	3.66E-01	4.83E-01	4.78E-01
1.05E+00	1.49E+00	1.40E-01	3.73E-01	4.93E-01	4.87E-01
1.07E+00	1.52E+00	1.42E-01	3.80E-01	5.02E-01	4.97E-01
1.09E+00	1.55E+00	1.45E-01	3.87E-01	5.12E-01	5.06E-01
1.11E+00	1.58E+00	1.48E-01	3.95E-01	5.22E-01	5.16E-01
1.13E+00	1.61E+00	1.51E-01	4.03E-01	5.32E-01	5.26E-01
1.15E+00	1.64E+00	1.54E-01	4.10E-01	5.42E-01	5.36E-01
1.17E+00	1.67E+00	1.57E-01	4.18E-01	5.53E-01	5.47E-01
1.19E+00	1.71E+00	1.60E-01	4.26E-01	5.64E-01	5.57E-01
1.21E+00	1.74E+00	1.63E-01	4.35E-01	5.74E-01	5.68E-01
1.23E+00	1.77E+00	1.66E-01	4.43E-01	5.86E-01	5.79E-01
1.25E+00	1.81E+00	1.69E-01	4.52E-01	5.97E-01	5.90E-01
1.27E+00	1.84E+00	1.72E-01	4.60E-01	6.08E-01	6.02E-01
1.29E+00	1.88E+00	1.76E-01	4.69E-01	6.20E-01	6.13E-01
1.31E+00	1.91E+00	1.79E-01	4.78E-01	6.32E-01	6.25E-01
1.33E+00	1.95E+00	1.83E-01	4.88E-01	6.45E-01	6.37E-01
1.35E+00	1.99E+00	1.86E-01	4.97E-01	6.57E-01	6.50E-01
1.37E+00	2.03E+00	1.90E-01	5.07E-01	6.70E-01	6.62E-01
1.39E+00	2.07E+00	1.93E-01	5.16E-01	6.83E-01	6.75E-01
1.41E+00	2.11E+00	1.97E-01	5.26E-01	6.96E-01	6.88E-01
1.43E+00	2.15E+00	2.01E-01	5.37E-01	7.09E-01	7.01E-01
1.45E+00	2.19E+00	2.05E-01	5.47E-01	7.23E-01	7.15E-01
1.47E+00	2.23E+00	2.09E-01	5.58E-01	7.37E-01	7.29E-01
1.49E+00	2.28E+00	2.13E-01	5.68E-01	7.51E-01	7.43E-01
1.51E+00	2.32E+00	2.17E-01	5.79E-01	7.66E-01	7.57E-01
1.53E+00	2.36E+00	2.21E-01	5.91E-01	7.81E-01	7.72E-01
1.55E+00	2.41E+00	2.25E-01	6.02E-01	7.96E-01	7.87E-01
1.57E+00	2.46E+00	2.30E-01	6.14E-01	8.11E-01	8.02E-01
1.59E+00	2.50E+00	2.34E-01	6.26E-01	8.27E-01	8.18E-01
1.61E+00	2.55E+00	2.39E-01	6.38E-01	8.43E-01	8.33E-01
1.63E+00	2.60E+00	2.43E-01	6.50E-01	8.59E-01	8.50E-01
1.65E+00	2.65E+00	2.48E-01	6.63E-01	8.76E-01	8.66E-01
1.67E+00	2.70E+00	2.53E-01	6.76E-01	8.93E-01	8.83E-01
1.69E+00	2.76E+00	2.58E-01	6.89E-01	9.10E-01	9.00E-01
1.71E+00	2.81E+00	2.63E-01	7.02E-01	9.28E-01	9.17E-01
1.73E+00	2.86E+00	2.68E-01	7.15E-01	9.46E-01	9.35E-01
1.75E+00	2.92E+00	2.73E-01	7.29E-01	9.64E-01	9.53E-01
1.77E+00	2.98E+00	2.78E-01	7.43E-01	9.83E-01	9.72E-01
1.79E+00	3.03E+00	2.84E-01	7.58E-01	1.00E+00	9.90E-01
1.81E+00	3.09E+00	2.89E-01	7.73E-01	1.02E+00	1.01E+00

Continued on next page

Table 3 – continued from previous page

E_x (MeV)	Total NLD (MeV^{-1})	J=0	J=1	J=2	J=3
1.83E+00	3.15E+00	2.95E-01	7.87E-01	1.04E+00	1.03E+00
1.85E+00	3.21E+00	3.00E-01	8.03E-01	1.06E+00	1.05E+00
1.87E+00	3.28E+00	3.06E-01	8.18E-01	1.08E+00	1.07E+00
1.89E+00	3.34E+00	3.12E-01	8.34E-01	1.10E+00	1.09E+00
1.91E+00	3.40E+00	3.18E-01	8.50E-01	1.12E+00	1.11E+00
1.93E+00	3.47E+00	3.24E-01	8.67E-01	1.15E+00	1.13E+00
1.95E+00	3.54E+00	3.31E-01	8.83E-01	1.17E+00	1.15E+00
1.97E+00	3.60E+00	3.37E-01	9.01E-01	1.19E+00	1.18E+00
1.99E+00	3.67E+00	3.44E-01	9.18E-01	1.21E+00	1.20E+00
2.01E+00	3.75E+00	3.50E-01	9.36E-01	1.24E+00	1.22E+00
2.03E+00	3.82E+00	3.57E-01	9.54E-01	1.26E+00	1.25E+00
2.05E+00	3.89E+00	3.64E-01	9.72E-01	1.29E+00	1.27E+00
2.07E+00	3.97E+00	3.71E-01	9.91E-01	1.31E+00	1.30E+00
2.09E+00	4.04E+00	3.78E-01	1.01E+00	1.34E+00	1.32E+00
2.11E+00	4.12E+00	3.86E-01	1.03E+00	1.36E+00	1.35E+00
2.13E+00	4.20E+00	3.93E-01	1.05E+00	1.39E+00	1.37E+00
2.15E+00	4.28E+00	4.01E-01	1.07E+00	1.41E+00	1.40E+00
2.17E+00	4.37E+00	4.08E-01	1.09E+00	1.44E+00	1.43E+00
2.19E+00	4.45E+00	4.16E-01	1.11E+00	1.47E+00	1.45E+00
2.21E+00	4.54E+00	4.24E-01	1.13E+00	1.50E+00	1.48E+00
2.23E+00	4.63E+00	4.32E-01	1.16E+00	1.53E+00	1.51E+00
2.25E+00	4.71E+00	4.41E-01	1.18E+00	1.56E+00	1.54E+00
2.27E+00	4.81E+00	4.49E-01	1.20E+00	1.59E+00	1.57E+00
2.29E+00	4.90E+00	4.58E-01	1.22E+00	1.62E+00	1.60E+00
2.31E+00	4.99E+00	4.67E-01	1.25E+00	1.65E+00	1.63E+00
2.33E+00	5.09E+00	4.76E-01	1.27E+00	1.68E+00	1.66E+00
2.35E+00	5.19E+00	4.85E-01	1.30E+00	1.71E+00	1.69E+00
2.37E+00	5.29E+00	4.95E-01	1.32E+00	1.75E+00	1.73E+00
2.39E+00	5.39E+00	5.04E-01	1.35E+00	1.78E+00	1.76E+00
2.41E+00	5.50E+00	5.14E-01	1.37E+00	1.81E+00	1.79E+00
2.43E+00	5.60E+00	5.24E-01	1.40E+00	1.85E+00	1.83E+00
2.45E+00	5.71E+00	5.34E-01	1.43E+00	1.89E+00	1.86E+00
2.47E+00	5.82E+00	5.44E-01	1.45E+00	1.92E+00	1.90E+00
2.49E+00	5.93E+00	5.55E-01	1.48E+00	1.96E+00	1.94E+00
2.51E+00	6.05E+00	5.66E-01	1.51E+00	2.00E+00	1.97E+00
2.53E+00	6.17E+00	5.77E-01	1.54E+00	2.04E+00	2.01E+00
2.55E+00	6.29E+00	5.88E-01	1.57E+00	2.08E+00	2.05E+00
2.57E+00	6.41E+00	5.99E-01	1.60E+00	2.12E+00	2.09E+00
2.59E+00	6.53E+00	6.11E-01	1.63E+00	2.16E+00	2.13E+00
2.61E+00	6.66E+00	6.23E-01	1.66E+00	2.20E+00	2.17E+00
2.63E+00	6.79E+00	6.35E-01	1.70E+00	2.24E+00	2.22E+00
2.65E+00	6.92E+00	6.47E-01	1.73E+00	2.28E+00	2.26E+00
2.67E+00	7.05E+00	6.59E-01	1.76E+00	2.33E+00	2.30E+00
2.69E+00	7.19E+00	6.72E-01	1.80E+00	2.37E+00	2.35E+00
2.71E+00	7.33E+00	6.85E-01	1.83E+00	2.42E+00	2.39E+00
2.73E+00	7.47E+00	6.98E-01	1.87E+00	2.47E+00	2.44E+00
2.75E+00	7.61E+00	7.12E-01	1.90E+00	2.51E+00	2.49E+00
2.77E+00	7.76E+00	7.26E-01	1.94E+00	2.56E+00	2.53E+00
2.79E+00	7.91E+00	7.40E-01	1.98E+00	2.61E+00	2.58E+00
2.81E+00	8.06E+00	7.54E-01	2.01E+00	2.66E+00	2.63E+00
2.83E+00	8.22E+00	7.69E-01	2.05E+00	2.71E+00	2.68E+00
2.85E+00	8.38E+00	7.84E-01	2.09E+00	2.77E+00	2.74E+00
2.87E+00	8.54E+00	7.99E-01	2.13E+00	2.82E+00	2.79E+00

Continued on next page

Table 3 – continued from previous page

E_x (MeV)	Total NLD (MeV^{-1})	J=0	J=1	J=2	J=3
2.89E+00	8.71E+00	8.14E-01	2.18E+00	2.88E+00	2.84E+00
2.91E+00	8.88E+00	8.30E-01	2.22E+00	2.93E+00	2.90E+00
2.93E+00	9.05E+00	8.46E-01	2.26E+00	2.99E+00	2.95E+00
2.95E+00	9.22E+00	8.62E-01	2.30E+00	3.05E+00	3.01E+00
2.97E+00	9.40E+00	8.79E-01	2.35E+00	3.10E+00	3.07E+00
2.99E+00	9.58E+00	8.96E-01	2.39E+00	3.16E+00	3.13E+00
3.01E+00	9.77E+00	9.13E-01	2.44E+00	3.23E+00	3.19E+00
3.03E+00	9.96E+00	9.31E-01	2.49E+00	3.29E+00	3.25E+00
3.05E+00	1.02E+01	9.49E-01	2.54E+00	3.35E+00	3.31E+00
3.07E+00	1.03E+01	9.68E-01	2.58E+00	3.42E+00	3.38E+00
3.09E+00	1.05E+01	9.86E-01	2.63E+00	3.48E+00	3.44E+00
3.11E+00	1.08E+01	1.01E+00	2.69E+00	3.55E+00	3.51E+00
3.13E+00	1.10E+01	1.02E+00	2.74E+00	3.62E+00	3.58E+00
3.15E+00	1.12E+01	1.04E+00	2.79E+00	3.69E+00	3.65E+00
3.17E+00	1.14E+01	1.06E+00	2.84E+00	3.76E+00	3.72E+00
3.19E+00	1.16E+01	1.09E+00	2.90E+00	3.83E+00	3.79E+00
3.21E+00	1.18E+01	1.11E+00	2.96E+00	3.91E+00	3.86E+00
3.23E+00	1.21E+01	1.13E+00	3.01E+00	3.98E+00	3.94E+00
3.25E+00	1.23E+01	1.15E+00	3.07E+00	4.06E+00	4.01E+00
3.27E+00	1.25E+01	1.17E+00	3.13E+00	4.14E+00	4.09E+00
3.29E+00	1.28E+01	1.19E+00	3.19E+00	4.22E+00	4.17E+00
3.31E+00	1.30E+01	1.22E+00	3.25E+00	4.30E+00	4.25E+00
3.33E+00	1.33E+01	1.24E+00	3.32E+00	4.38E+00	4.33E+00
3.35E+00	1.35E+01	1.27E+00	3.38E+00	4.47E+00	4.42E+00
3.37E+00	1.38E+01	1.29E+00	3.45E+00	4.55E+00	4.50E+00
3.39E+00	1.41E+01	1.31E+00	3.51E+00	4.64E+00	4.59E+00
3.41E+00	1.43E+01	1.34E+00	3.58E+00	4.73E+00	4.68E+00
3.43E+00	1.46E+01	1.37E+00	3.65E+00	4.82E+00	4.77E+00
3.45E+00	1.49E+01	1.39E+00	3.72E+00	4.92E+00	4.86E+00
3.47E+00	1.52E+01	1.42E+00	3.79E+00	5.01E+00	4.96E+00
3.49E+00	1.55E+01	1.45E+00	3.87E+00	5.11E+00	5.05E+00
3.51E+00	1.58E+01	1.48E+00	3.94E+00	5.21E+00	5.15E+00
3.53E+00	1.61E+01	1.50E+00	4.02E+00	5.31E+00	5.25E+00
3.55E+00	1.64E+01	1.53E+00	4.09E+00	5.41E+00	5.35E+00
3.57E+00	1.67E+01	1.56E+00	4.17E+00	5.52E+00	5.45E+00
3.59E+00	1.70E+01	1.59E+00	4.25E+00	5.62E+00	5.56E+00
3.61E+00	1.74E+01	1.62E+00	4.34E+00	5.73E+00	5.67E+00
3.63E+00	1.77E+01	1.65E+00	4.42E+00	5.84E+00	5.78E+00
3.65E+00	1.80E+01	1.69E+00	4.51E+00	5.96E+00	5.89E+00
3.67E+00	1.84E+01	1.72E+00	4.59E+00	6.07E+00	6.00E+00
3.69E+00	1.87E+01	1.75E+00	4.68E+00	6.19E+00	6.12E+00
3.71E+00	1.91E+01	1.79E+00	4.77E+00	6.31E+00	6.24E+00
3.73E+00	1.95E+01	1.82E+00	4.87E+00	6.43E+00	6.36E+00
3.75E+00	1.99E+01	1.86E+00	4.96E+00	6.56E+00	6.48E+00
3.77E+00	2.02E+01	1.89E+00	5.06E+00	6.68E+00	6.61E+00
3.79E+00	2.06E+01	1.93E+00	5.15E+00	6.81E+00	6.74E+00
3.81E+00	2.10E+01	1.97E+00	5.25E+00	6.94E+00	6.87E+00
3.83E+00	2.14E+01	2.00E+00	5.36E+00	7.08E+00	7.00E+00
3.85E+00	2.19E+01	2.04E+00	5.46E+00	7.22E+00	7.13E+00
3.87E+00	2.23E+01	2.08E+00	5.56E+00	7.36E+00	7.27E+00
3.89E+00	2.27E+01	2.12E+00	5.67E+00	7.50E+00	7.41E+00
3.91E+00	2.31E+01	2.16E+00	5.78E+00	7.64E+00	7.56E+00
3.93E+00	2.36E+01	2.21E+00	5.89E+00	7.79E+00	7.70E+00

Continued on next page

Table 3 – continued from previous page

E_x (MeV)	Total NLD (MeV^{-1})	J=0	J=1	J=2	J=3
3.95E+00	2.41E+01	2.25E+00	6.01E+00	7.94E+00	7.85E+00
3.97E+00	2.45E+01	2.29E+00	6.12E+00	8.10E+00	8.00E+00
3.99E+00	2.50E+01	2.34E+00	6.24E+00	8.25E+00	8.16E+00
4.01E+00	2.55E+01	2.38E+00	6.36E+00	8.41E+00	8.32E+00
4.03E+00	2.60E+01	2.43E+00	6.49E+00	8.57E+00	8.48E+00
4.05E+00	2.65E+01	2.48E+00	6.61E+00	8.74E+00	8.64E+00
4.07E+00	2.70E+01	2.52E+00	6.74E+00	8.91E+00	8.81E+00
4.09E+00	2.75E+01	2.57E+00	6.87E+00	9.08E+00	8.98E+00
4.11E+00	2.80E+01	2.62E+00	7.00E+00	9.26E+00	9.15E+00
4.13E+00	2.86E+01	2.67E+00	7.14E+00	9.44E+00	9.33E+00
4.15E+00	2.91E+01	2.72E+00	7.28E+00	9.62E+00	9.51E+00
4.17E+00	2.97E+01	2.78E+00	7.42E+00	9.81E+00	9.70E+00
4.19E+00	3.03E+01	2.83E+00	7.56E+00	1.00E+01	9.88E+00
4.21E+00	3.09E+01	2.89E+00	7.71E+00	1.02E+01	1.01E+01
4.23E+00	3.15E+01	2.94E+00	7.86E+00	1.04E+01	1.03E+01
4.25E+00	3.21E+01	3.00E+00	8.01E+00	1.06E+01	1.05E+01
4.27E+00	3.27E+01	3.06E+00	8.17E+00	1.08E+01	1.07E+01
4.29E+00	3.33E+01	3.12E+00	8.32E+00	1.10E+01	1.09E+01
4.31E+00	3.40E+01	3.18E+00	8.48E+00	1.12E+01	1.11E+01
4.33E+00	3.46E+01	3.24E+00	8.65E+00	1.14E+01	1.13E+01
4.35E+00	3.53E+01	3.30E+00	8.82E+00	1.17E+01	1.15E+01
4.37E+00	3.60E+01	3.36E+00	8.99E+00	1.19E+01	1.17E+01
4.39E+00	3.67E+01	3.43E+00	9.16E+00	1.21E+01	1.20E+01
4.41E+00	3.74E+01	3.50E+00	9.34E+00	1.23E+01	1.22E+01
4.43E+00	3.81E+01	3.56E+00	9.52E+00	1.26E+01	1.24E+01
4.45E+00	3.88E+01	3.63E+00	9.70E+00	1.28E+01	1.27E+01
4.47E+00	3.96E+01	3.70E+00	9.89E+00	1.31E+01	1.29E+01
4.49E+00	4.04E+01	3.77E+00	1.01E+01	1.33E+01	1.32E+01
4.51E+00	4.11E+01	3.85E+00	1.03E+01	1.36E+01	1.34E+01
4.53E+00	4.19E+01	3.92E+00	1.05E+01	1.38E+01	1.37E+01
4.55E+00	4.27E+01	4.00E+00	1.07E+01	1.41E+01	1.40E+01
4.57E+00	4.36E+01	4.07E+00	1.09E+01	1.44E+01	1.42E+01
4.59E+00	4.44E+01	4.15E+00	1.11E+01	1.47E+01	1.45E+01
4.61E+00	4.53E+01	4.23E+00	1.13E+01	1.50E+01	1.48E+01
4.63E+00	4.62E+01	4.32E+00	1.15E+01	1.52E+01	1.51E+01
4.65E+00	4.70E+01	4.40E+00	1.18E+01	1.55E+01	1.54E+01
4.67E+00	4.80E+01	4.48E+00	1.20E+01	1.58E+01	1.57E+01
4.69E+00	4.89E+01	4.57E+00	1.22E+01	1.61E+01	1.60E+01
4.71E+00	4.98E+01	4.66E+00	1.24E+01	1.65E+01	1.63E+01
4.73E+00	5.08E+01	4.75E+00	1.27E+01	1.68E+01	1.66E+01
4.75E+00	5.18E+01	4.84E+00	1.29E+01	1.71E+01	1.69E+01
4.77E+00	5.28E+01	4.94E+00	1.32E+01	1.74E+01	1.72E+01
4.79E+00	5.38E+01	5.03E+00	1.34E+01	1.78E+01	1.76E+01
4.81E+00	5.48E+01	5.13E+00	1.37E+01	1.81E+01	1.79E+01
4.83E+00	5.59E+01	5.23E+00	1.40E+01	1.85E+01	1.83E+01
4.85E+00	5.70E+01	5.33E+00	1.42E+01	1.88E+01	1.86E+01
4.87E+00	5.81E+01	5.43E+00	1.45E+01	1.92E+01	1.90E+01
4.89E+00	5.92E+01	5.54E+00	1.48E+01	1.96E+01	1.93E+01
4.91E+00	6.04E+01	5.64E+00	1.51E+01	1.99E+01	1.97E+01
4.93E+00	6.15E+01	5.75E+00	1.54E+01	2.03E+01	2.01E+01
4.95E+00	6.27E+01	5.87E+00	1.57E+01	2.07E+01	2.05E+01
4.97E+00	6.39E+01	5.98E+00	1.60E+01	2.11E+01	2.09E+01
4.99E+00	6.52E+01	6.09E+00	1.63E+01	2.15E+01	2.13E+01

Continued on next page

Table 3 – continued from previous page

E_x (MeV)	Total NLD (MeV^{-1})	J=0	J=1	J=2	J=3
5.01E+00	6.64E+01	6.21E+00	1.66E+01	2.19E+01	2.17E+01
5.03E+00	6.77E+01	6.33E+00	1.69E+01	2.24E+01	2.21E+01
5.05E+00	6.90E+01	6.46E+00	1.72E+01	2.28E+01	2.25E+01
5.07E+00	7.04E+01	6.58E+00	1.76E+01	2.32E+01	2.30E+01
5.09E+00	7.17E+01	6.71E+00	1.79E+01	2.37E+01	2.34E+01
5.11E+00	7.31E+01	6.84E+00	1.83E+01	2.41E+01	2.39E+01
5.13E+00	7.45E+01	6.97E+00	1.86E+01	2.46E+01	2.43E+01
5.15E+00	7.60E+01	7.10E+00	1.90E+01	2.51E+01	2.48E+01
5.17E+00	7.74E+01	7.24E+00	1.93E+01	2.56E+01	2.53E+01
5.19E+00	7.89E+01	7.38E+00	1.97E+01	2.61E+01	2.58E+01
5.21E+00	8.05E+01	7.53E+00	2.01E+01	2.66E+01	2.63E+01
5.23E+00	8.20E+01	7.67E+00	2.05E+01	2.71E+01	2.68E+01
5.25E+00	8.36E+01	7.82E+00	2.09E+01	2.76E+01	2.73E+01
5.27E+00	8.52E+01	7.97E+00	2.13E+01	2.81E+01	2.78E+01
5.29E+00	8.69E+01	8.12E+00	2.17E+01	2.87E+01	2.84E+01
5.31E+00	8.86E+01	8.28E+00	2.21E+01	2.92E+01	2.89E+01
5.33E+00	9.03E+01	8.44E+00	2.26E+01	2.98E+01	2.95E+01
5.35E+00	9.20E+01	8.61E+00	2.30E+01	3.04E+01	3.00E+01
5.37E+00	9.38E+01	8.77E+00	2.34E+01	3.10E+01	3.06E+01
5.39E+00	9.56E+01	8.94E+00	2.39E+01	3.16E+01	3.12E+01
5.41E+00	9.75E+01	9.12E+00	2.44E+01	3.22E+01	3.18E+01
5.43E+00	9.94E+01	9.29E+00	2.48E+01	3.28E+01	3.24E+01
5.45E+00	1.01E+02	9.47E+00	2.53E+01	3.34E+01	3.31E+01
5.47E+00	1.03E+02	9.65E+00	2.58E+01	3.41E+01	3.37E+01
5.49E+00	1.05E+02	9.84E+00	2.63E+01	3.48E+01	3.44E+01
5.51E+00	1.07E+02	1.00E+01	2.68E+01	3.54E+01	3.50E+01
5.53E+00	1.09E+02	1.02E+01	2.73E+01	3.61E+01	3.57E+01
5.55E+00	1.11E+02	1.04E+01	2.78E+01	3.68E+01	3.64E+01
5.57E+00	1.14E+02	1.06E+01	2.84E+01	3.75E+01	3.71E+01
5.59E+00	1.16E+02	1.08E+01	2.89E+01	3.82E+01	3.78E+01
5.61E+00	1.18E+02	1.10E+01	2.95E+01	3.90E+01	3.85E+01
5.63E+00	1.20E+02	1.13E+01	3.01E+01	3.97E+01	3.93E+01
5.65E+00	1.23E+02	1.15E+01	3.07E+01	4.05E+01	4.01E+01
5.67E+00	1.25E+02	1.17E+01	3.12E+01	4.13E+01	4.08E+01
5.69E+00	1.27E+02	1.19E+01	3.18E+01	4.21E+01	4.16E+01
5.71E+00	1.30E+02	1.22E+01	3.25E+01	4.29E+01	4.24E+01
5.73E+00	1.32E+02	1.24E+01	3.31E+01	4.37E+01	4.32E+01
5.75E+00	1.35E+02	1.26E+01	3.37E+01	4.46E+01	4.41E+01
5.77E+00	1.38E+02	1.29E+01	3.44E+01	4.55E+01	4.49E+01
5.79E+00	1.40E+02	1.31E+01	3.51E+01	4.63E+01	4.58E+01
5.81E+00	1.43E+02	1.34E+01	3.57E+01	4.72E+01	4.67E+01
5.83E+00	1.46E+02	1.36E+01	3.64E+01	4.81E+01	4.76E+01
5.85E+00	1.49E+02	1.39E+01	3.71E+01	4.91E+01	4.85E+01
5.87E+00	1.51E+02	1.42E+01	3.78E+01	5.00E+01	4.95E+01
5.89E+00	1.54E+02	1.44E+01	3.86E+01	5.10E+01	5.04E+01
5.91E+00	1.57E+02	1.47E+01	3.93E+01	5.20E+01	5.14E+01
5.93E+00	1.60E+02	1.50E+01	4.01E+01	5.30E+01	5.24E+01
5.95E+00	1.64E+02	1.53E+01	4.09E+01	5.40E+01	5.34E+01
5.97E+00	1.67E+02	1.56E+01	4.17E+01	5.51E+01	5.44E+01
5.99E+00	1.70E+02	1.59E+01	4.25E+01	5.61E+01	5.55E+01
6.01E+00	1.73E+02	1.62E+01	4.33E+01	5.72E+01	5.66E+01
6.03E+00	1.77E+02	1.65E+01	4.41E+01	5.83E+01	5.77E+01
6.05E+00	1.80E+02	1.68E+01	4.50E+01	5.94E+01	5.88E+01

Continued on next page

Table 3 – continued from previous page

E_x (MeV)	Total NLD (MeV^{-1})	J=0	J=1	J=2	J=3
6.07E+00	1.84E+02	1.72E+01	4.58E+01	6.06E+01	5.99E+01
6.09E+00	1.87E+02	1.75E+01	4.67E+01	6.18E+01	6.11E+01
6.11E+00	1.91E+02	1.78E+01	4.76E+01	6.30E+01	6.23E+01
6.13E+00	1.94E+02	1.82E+01	4.86E+01	6.42E+01	6.35E+01
6.15E+00	1.98E+02	1.85E+01	4.95E+01	6.54E+01	6.47E+01
6.17E+00	2.02E+02	1.89E+01	5.05E+01	6.67E+01	6.59E+01
6.19E+00	2.06E+02	1.93E+01	5.14E+01	6.80E+01	6.72E+01
6.21E+00	2.10E+02	1.96E+01	5.24E+01	6.93E+01	6.85E+01
6.23E+00	2.14E+02	2.00E+01	5.34E+01	7.06E+01	6.98E+01
6.25E+00	2.18E+02	2.04E+01	5.45E+01	7.20E+01	7.12E+01
6.27E+00	2.22E+02	2.08E+01	5.55E+01	7.34E+01	7.26E+01
6.29E+00	2.27E+02	2.12E+01	5.66E+01	7.48E+01	7.40E+01
6.31E+00	2.31E+02	2.16E+01	5.77E+01	7.63E+01	7.54E+01
6.33E+00	2.35E+02	2.20E+01	5.88E+01	7.77E+01	7.69E+01
6.35E+00	2.40E+02	2.24E+01	6.00E+01	7.92E+01	7.84E+01
6.37E+00	2.45E+02	2.29E+01	6.11E+01	8.08E+01	7.99E+01
6.39E+00	2.49E+02	2.33E+01	6.23E+01	8.23E+01	8.14E+01
6.41E+00	2.54E+02	2.38E+01	6.35E+01	8.39E+01	8.30E+01
6.43E+00	2.59E+02	2.42E+01	6.47E+01	8.56E+01	8.46E+01
6.45E+00	2.64E+02	2.47E+01	6.60E+01	8.72E+01	8.62E+01
6.47E+00	2.69E+02	2.52E+01	6.73E+01	8.89E+01	8.79E+01
6.49E+00	2.74E+02	2.57E+01	6.86E+01	9.06E+01	8.96E+01
6.51E+00	2.80E+02	2.62E+01	6.99E+01	9.24E+01	9.13E+01
6.53E+00	2.85E+02	2.67E+01	7.12E+01	9.42E+01	9.31E+01
6.55E+00	2.91E+02	2.72E+01	7.26E+01	9.60E+01	9.49E+01
6.57E+00	2.96E+02	2.77E+01	7.40E+01	9.79E+01	9.67E+01
6.59E+00	3.02E+02	2.82E+01	7.55E+01	9.97E+01	9.86E+01
6.61E+00	3.08E+02	2.88E+01	7.69E+01	1.02E+02	1.01E+02
6.63E+00	3.14E+02	2.94E+01	7.84E+01	1.04E+02	1.02E+02
6.65E+00	3.20E+02	2.99E+01	7.99E+01	1.06E+02	1.04E+02
6.67E+00	3.26E+02	3.05E+01	8.15E+01	1.08E+02	1.06E+02
6.69E+00	3.32E+02	3.11E+01	8.31E+01	1.10E+02	1.09E+02
6.71E+00	3.39E+02	3.17E+01	8.47E+01	1.12E+02	1.11E+02
6.73E+00	3.45E+02	3.23E+01	8.63E+01	1.14E+02	1.13E+02
6.75E+00	3.52E+02	3.29E+01	8.80E+01	1.16E+02	1.15E+02
6.77E+00	3.59E+02	3.36E+01	8.97E+01	1.19E+02	1.17E+02
6.79E+00	3.66E+02	3.42E+01	9.14E+01	1.21E+02	1.19E+02
6.81E+00	3.73E+02	3.49E+01	9.32E+01	1.23E+02	1.22E+02
6.83E+00	3.80E+02	3.56E+01	9.50E+01	1.26E+02	1.24E+02
6.85E+00	3.88E+02	3.62E+01	9.68E+01	1.28E+02	1.27E+02
6.87E+00	3.95E+02	3.69E+01	9.87E+01	1.30E+02	1.29E+02
6.89E+00	4.03E+02	3.77E+01	1.01E+02	1.33E+02	1.31E+02
6.91E+00	4.11E+02	3.84E+01	1.03E+02	1.36E+02	1.34E+02
6.93E+00	4.18E+02	3.91E+01	1.05E+02	1.38E+02	1.37E+02
6.95E+00	4.27E+02	3.99E+01	1.07E+02	1.41E+02	1.39E+02
6.97E+00	4.35E+02	4.07E+01	1.09E+02	1.44E+02	1.42E+02
6.99E+00	4.43E+02	4.14E+01	1.11E+02	1.46E+02	1.45E+02
7.01E+00	4.52E+02	4.22E+01	1.13E+02	1.49E+02	1.48E+02
7.03E+00	4.61E+02	4.31E+01	1.15E+02	1.52E+02	1.50E+02
7.05E+00	4.69E+02	4.39E+01	1.17E+02	1.55E+02	1.53E+02
7.07E+00	4.79E+02	4.48E+01	1.20E+02	1.58E+02	1.56E+02
7.09E+00	4.88E+02	4.56E+01	1.22E+02	1.61E+02	1.59E+02
7.11E+00	4.97E+02	4.65E+01	1.24E+02	1.64E+02	1.62E+02

Continued on next page

Table 3 – continued from previous page

E_x (MeV)	Total NLD (MeV^{-1})	J=0	J=1	J=2	J=3
7.13E+00	5.07E+02	4.74E+01	1.27E+02	1.67E+02	1.65E+02
7.15E+00	5.17E+02	4.83E+01	1.29E+02	1.71E+02	1.69E+02
7.17E+00	5.27E+02	4.93E+01	1.32E+02	1.74E+02	1.72E+02
7.19E+00	5.37E+02	5.02E+01	1.34E+02	1.77E+02	1.75E+02
7.21E+00	5.47E+02	5.12E+01	1.37E+02	1.81E+02	1.79E+02
7.23E+00	5.58E+02	5.22E+01	1.39E+02	1.84E+02	1.82E+02
7.25E+00	5.69E+02	5.32E+01	1.42E+02	1.88E+02	1.86E+02
7.27E+00	5.80E+02	5.42E+01	1.45E+02	1.91E+02	1.89E+02
7.29E+00	5.91E+02	5.53E+01	1.48E+02	1.95E+02	1.93E+02
7.31E+00	6.02E+02	5.63E+01	1.50E+02	1.99E+02	1.97E+02
7.33E+00	6.14E+02	5.74E+01	1.53E+02	2.03E+02	2.00E+02
7.35E+00	6.26E+02	5.85E+01	1.56E+02	2.07E+02	2.04E+02
7.37E+00	6.38E+02	5.97E+01	1.59E+02	2.11E+02	2.08E+02
7.39E+00	6.50E+02	6.08E+01	1.62E+02	2.15E+02	2.12E+02
7.41E+00	6.63E+02	6.20E+01	1.66E+02	2.19E+02	2.16E+02
7.43E+00	6.76E+02	6.32E+01	1.69E+02	2.23E+02	2.21E+02
7.45E+00	6.89E+02	6.44E+01	1.72E+02	2.27E+02	2.25E+02
7.47E+00	7.02E+02	6.57E+01	1.75E+02	2.32E+02	2.29E+02
7.49E+00	7.16E+02	6.69E+01	1.79E+02	2.36E+02	2.34E+02
7.51E+00	7.30E+02	6.82E+01	1.82E+02	2.41E+02	2.38E+02
7.53E+00	7.44E+02	6.95E+01	1.86E+02	2.46E+02	2.43E+02
7.55E+00	7.58E+02	7.09E+01	1.89E+02	2.50E+02	2.48E+02
7.57E+00	7.73E+02	7.23E+01	1.93E+02	2.55E+02	2.52E+02
7.59E+00	7.88E+02	7.37E+01	1.97E+02	2.60E+02	2.57E+02
7.61E+00	8.03E+02	7.51E+01	2.01E+02	2.65E+02	2.62E+02
7.63E+00	8.19E+02	7.65E+01	2.04E+02	2.70E+02	2.67E+02
7.65E+00	8.34E+02	7.80E+01	2.08E+02	2.76E+02	2.72E+02
7.67E+00	8.51E+02	7.95E+01	2.12E+02	2.81E+02	2.78E+02
7.69E+00	8.67E+02	8.11E+01	2.17E+02	2.86E+02	2.83E+02
7.71E+00	8.84E+02	8.26E+01	2.21E+02	2.92E+02	2.89E+02
7.73E+00	9.01E+02	8.42E+01	2.25E+02	2.97E+02	2.94E+02
7.75E+00	9.18E+02	8.59E+01	2.29E+02	3.03E+02	3.00E+02
7.77E+00	9.36E+02	8.75E+01	2.34E+02	3.09E+02	3.06E+02
7.79E+00	9.54E+02	8.92E+01	2.38E+02	3.15E+02	3.12E+02
7.81E+00	9.73E+02	9.10E+01	2.43E+02	3.21E+02	3.18E+02
7.83E+00	9.92E+02	9.27E+01	2.48E+02	3.27E+02	3.24E+02
7.85E+00	1.01E+03	9.45E+01	2.53E+02	3.34E+02	3.30E+02
7.87E+00	1.03E+03	9.63E+01	2.57E+02	3.40E+02	3.36E+02
7.89E+00	1.05E+03	9.82E+01	2.62E+02	3.47E+02	3.43E+02
7.91E+00	1.07E+03	1.00E+02	2.67E+02	3.54E+02	3.50E+02
7.93E+00	1.09E+03	1.02E+02	2.73E+02	3.60E+02	3.56E+02
7.95E+00	1.11E+03	1.04E+02	2.78E+02	3.67E+02	3.63E+02
7.97E+00	1.13E+03	1.06E+02	2.83E+02	3.74E+02	3.70E+02
7.99E+00	1.16E+03	1.08E+02	2.89E+02	3.82E+02	3.77E+02
8.01E+00	1.18E+03	1.10E+02	2.94E+02	3.89E+02	3.85E+02
8.03E+00	1.20E+03	1.12E+02	3.00E+02	3.97E+02	3.92E+02
8.05E+00	1.22E+03	1.14E+02	3.06E+02	4.04E+02	4.00E+02
8.07E+00	1.25E+03	1.17E+02	3.12E+02	4.12E+02	4.07E+02
8.09E+00	1.27E+03	1.19E+02	3.18E+02	4.20E+02	4.15E+02
8.11E+00	1.30E+03	1.21E+02	3.24E+02	4.28E+02	4.23E+02
8.13E+00	1.32E+03	1.24E+02	3.30E+02	4.36E+02	4.32E+02
8.15E+00	1.35E+03	1.26E+02	3.37E+02	4.45E+02	4.40E+02
8.17E+00	1.37E+03	1.28E+02	3.43E+02	4.54E+02	4.48E+02

Continued on next page

Table 3 – continued from previous page

E_x (MeV)	Total NLD (MeV^{-1})	J=0	J=1	J=2	J=3
8.19E+00	1.40E+03	1.31E+02	3.50E+02	4.62E+02	4.57E+02
8.21E+00	1.43E+03	1.33E+02	3.57E+02	4.71E+02	4.66E+02
8.23E+00	1.45E+03	1.36E+02	3.63E+02	4.80E+02	4.75E+02
8.25E+00	1.48E+03	1.39E+02	3.70E+02	4.90E+02	4.84E+02

Table 4: EMPIRE default NLD for ^{74}Zn including spin-dependent NLD for J=0-5. An equal parity distribution is assumed.

E_x (MeV)	Total NLD (MeV^{-1})	J=0	J=1	J=2	J=3	J=4	J=5
0.000	0.00E+00	0.00E+00	0.00E+00	0.00E+00	0.00E+00	0.00E+00	0.00E+00
0.106	8.86E-02	3.73E-02	4.13E-02	9.39E-03	6.63E-04	1.59E-05	1.35E-07
0.212	9.59E-02	2.97E-02	4.44E-02	1.84E-02	3.18E-03	2.52E-04	9.52E-06
0.318	1.16E-01	2.93E-02	5.03E-02	2.75E-02	7.25E-03	1.01E-03	7.62E-05
0.424	1.44E-01	3.13E-02	5.85E-02	3.80E-02	1.29E-02	2.52E-03	2.93E-04
0.530	1.80E-01	3.47E-02	6.89E-02	5.03E-02	2.05E-02	5.06E-03	7.89E-04
0.637	2.27E-01	3.93E-02	8.15E-02	6.50E-02	3.02E-02	8.90E-03	1.73E-03
0.743	2.85E-01	4.51E-02	9.68E-02	8.27E-02	4.26E-02	1.44E-02	3.32E-03
0.849	3.58E-01	5.21E-02	1.15E-01	1.04E-01	5.81E-02	2.20E-02	5.83E-03
0.955	4.49E-01	6.06E-02	1.37E-01	1.30E-01	7.75E-02	3.22E-02	9.56E-03
1.061	5.60E-01	7.06E-02	1.63E-01	1.60E-01	1.02E-01	4.55E-02	1.49E-02
1.167	6.96E-01	8.25E-02	1.93E-01	1.97E-01	1.31E-01	6.28E-02	2.24E-02
1.273	8.63E-01	9.65E-02	2.29E-01	2.40E-01	1.67E-01	8.49E-02	3.25E-02
1.379	1.07E+00	1.13E-01	2.72E-01	2.92E-01	2.11E-01	1.13E-01	4.60E-02
1.485	1.31E+00	1.32E-01	3.22E-01	3.53E-01	2.65E-01	1.48E-01	6.37E-02
1.591	1.61E+00	1.55E-01	3.80E-01	4.26E-01	3.29E-01	1.91E-01	8.67E-02
1.697	1.97E+00	1.81E-01	4.49E-01	5.12E-01	4.06E-01	2.45E-01	1.16E-01
1.803	2.40E+00	2.11E-01	5.29E-01	6.13E-01	4.98E-01	3.11E-01	1.54E-01
1.909	2.93E+00	2.47E-01	6.22E-01	7.33E-01	6.09E-01	3.91E-01	2.01E-01
2.016	3.55E+00	2.88E-01	7.31E-01	8.73E-01	7.40E-01	4.88E-01	2.59E-01
2.122	4.30E+00	3.36E-01	8.58E-01	1.04E+00	8.97E-01	6.06E-01	3.32E-01
2.228	5.19E+00	3.91E-01	1.01E+00	1.23E+00	1.08E+00	7.49E-01	4.22E-01
2.334	6.25E+00	4.56E-01	1.18E+00	1.45E+00	1.30E+00	9.19E-01	5.33E-01
2.440	7.51E+00	5.30E-01	1.38E+00	1.72E+00	1.56E+00	1.12E+00	6.67E-01
2.546	9.01E+00	6.16E-01	1.61E+00	2.02E+00	1.86E+00	1.37E+00	8.31E-01
2.652	1.08E+01	7.15E-01	1.87E+00	2.38E+00	2.22E+00	1.66E+00	1.03E+00
2.758	1.29E+01	8.29E-01	2.18E+00	2.79E+00	2.64E+00	2.00E+00	1.27E+00
2.864	1.54E+01	9.60E-01	2.54E+00	3.27E+00	3.12E+00	2.41E+00	1.56E+00
2.970	1.83E+01	1.11E+00	2.95E+00	3.83E+00	3.69E+00	2.89E+00	1.90E+00
3.076	2.17E+01	1.29E+00	3.42E+00	4.47E+00	4.36E+00	3.46E+00	2.31E+00
3.182	2.58E+01	1.49E+00	3.96E+00	5.22E+00	5.13E+00	4.12E+00	2.80E+00
3.288	3.05E+01	1.72E+00	4.59E+00	6.08E+00	6.03E+00	4.91E+00	3.38E+00
3.395	3.61E+01	1.98E+00	5.31E+00	7.07E+00	7.08E+00	5.82E+00	4.07E+00
3.501	4.26E+01	2.28E+00	6.13E+00	8.22E+00	8.29E+00	6.89E+00	4.89E+00
3.607	5.02E+01	2.63E+00	7.08E+00	9.53E+00	9.70E+00	8.14E+00	5.85E+00
3.713	5.91E+01	3.02E+00	8.17E+00	1.11E+01	1.13E+01	9.60E+00	6.98E+00
3.819	6.95E+01	3.47E+00	9.41E+00	1.28E+01	1.32E+01	1.13E+01	8.30E+00

Continued on next page

Table 4 – continued from previous page

E_x (MeV)	Total NLD (MeV^{-1})	J=0	J=1	J=2	J=3	J=4	J=5
3.925	8.15E+01	3.99E+00	1.08E+01	1.48E+01	1.54E+01	1.33E+01	9.86E+00
4.031	9.56E+01	4.58E+00	1.25E+01	1.71E+01	1.79E+01	1.56E+01	1.17E+01
4.137	1.12E+02	5.25E+00	1.43E+01	1.97E+01	2.07E+01	1.82E+01	1.38E+01
4.243	1.31E+02	6.02E+00	1.64E+01	2.27E+01	2.40E+01	2.13E+01	1.63E+01
4.349	1.53E+02	6.89E+00	1.89E+01	2.62E+01	2.78E+01	2.48E+01	1.92E+01
4.455	1.78E+02	7.89E+00	2.16E+01	3.01E+01	3.22E+01	2.89E+01	2.26E+01
4.561	2.08E+02	9.02E+00	2.48E+01	3.46E+01	3.72E+01	3.36E+01	2.65E+01
4.668	2.42E+02	1.03E+01	2.84E+01	3.98E+01	4.30E+01	3.91E+01	3.10E+01
4.774	2.82E+02	1.18E+01	3.25E+01	4.56E+01	4.95E+01	4.53E+01	3.63E+01
4.880	3.27E+02	1.34E+01	3.71E+01	5.23E+01	5.70E+01	5.26E+01	4.24E+01
4.986	3.80E+02	1.53E+01	4.24E+01	6.00E+01	6.56E+01	6.08E+01	4.94E+01
5.092	4.41E+02	1.75E+01	4.84E+01	6.86E+01	7.55E+01	7.03E+01	5.75E+01
5.198	5.10E+02	1.99E+01	5.52E+01	7.85E+01	8.67E+01	8.12E+01	6.69E+01
5.304	5.91E+02	2.27E+01	6.29E+01	8.98E+01	9.95E+01	9.37E+01	7.76E+01
5.410	6.83E+02	2.58E+01	7.17E+01	1.03E+02	1.14E+02	1.08E+02	9.01E+01
5.516	7.90E+02	2.94E+01	8.17E+01	1.17E+02	1.31E+02	1.24E+02	1.04E+02
5.622	9.12E+02	3.34E+01	9.29E+01	1.34E+02	1.50E+02	1.43E+02	1.21E+02
5.728	1.05E+03	3.79E+01	1.06E+02	1.52E+02	1.71E+02	1.64E+02	1.40E+02
5.834	1.21E+03	4.31E+01	1.20E+02	1.74E+02	1.96E+02	1.89E+02	1.61E+02
5.940	1.40E+03	4.89E+01	1.37E+02	1.98E+02	2.24E+02	2.17E+02	1.86E+02
6.047	1.61E+03	5.54E+01	1.55E+02	2.25E+02	2.55E+02	2.48E+02	2.14E+02
6.153	1.85E+03	6.29E+01	1.76E+02	2.56E+02	2.91E+02	2.85E+02	2.47E+02
6.259	2.13E+03	7.13E+01	2.00E+02	2.91E+02	3.32E+02	3.26E+02	2.84E+02
6.365	2.45E+03	8.08E+01	2.27E+02	3.30E+02	3.79E+02	3.73E+02	3.26E+02
6.471	2.79E+03	9.08E+01	2.55E+02	3.73E+02	4.28E+02	4.23E+02	3.72E+02
6.577	3.11E+03	9.99E+01	2.81E+02	4.11E+02	4.74E+02	4.70E+02	4.15E+02
6.683	3.47E+03	1.10E+02	3.09E+02	4.53E+02	5.23E+02	5.20E+02	4.62E+02
6.789	3.85E+03	1.21E+02	3.40E+02	4.98E+02	5.77E+02	5.76E+02	5.12E+02
6.895	4.27E+03	1.32E+02	3.72E+02	5.47E+02	6.35E+02	6.35E+02	5.68E+02
7.001	4.72E+03	1.45E+02	4.08E+02	6.00E+02	6.98E+02	7.00E+02	6.28E+02
7.107	5.22E+03	1.58E+02	4.46E+02	6.57E+02	7.66E+02	7.70E+02	6.93E+02
7.213	5.75E+03	1.72E+02	4.87E+02	7.18E+02	8.39E+02	8.46E+02	7.64E+02
7.319	6.33E+03	1.88E+02	5.30E+02	7.84E+02	9.17E+02	9.27E+02	8.40E+02
7.426	6.96E+03	2.04E+02	5.77E+02	8.54E+02	1.00E+03	1.02E+03	9.22E+02
7.532	7.63E+03	2.22E+02	6.27E+02	9.29E+02	1.09E+03	1.11E+03	1.01E+03
7.638	8.36E+03	2.40E+02	6.80E+02	1.01E+03	1.19E+03	1.21E+03	1.11E+03
7.744	9.14E+03	2.60E+02	7.37E+02	1.09E+03	1.29E+03	1.32E+03	1.21E+03
7.850	9.97E+03	2.81E+02	7.97E+02	1.19E+03	1.40E+03	1.43E+03	1.32E+03

Continued on next page

Table 4 – continued from previous page

E_x (MeV)	Total NLD (MeV^{-1})	J=0	J=1	J=2	J=3	J=4	J=5
7.956	1.09E+04	3.03E+02	8.60E+02	1.28E+03	1.51E+03	1.55E+03	1.43E+03
8.062	1.18E+04	3.27E+02	9.27E+02	1.38E+03	1.64E+03	1.68E+03	1.55E+03
8.168	1.28E+04	3.52E+02	9.98E+02	1.49E+03	1.77E+03	1.82E+03	1.68E+03
8.274	1.39E+04	3.78E+02	1.07E+03	1.60E+03	1.90E+03	1.96E+03	1.82E+03
8.380	1.50E+04	4.05E+02	1.15E+03	1.72E+03	2.05E+03	2.12E+03	1.97E+03

Table 5: EMPIRE default NLD for ^{74}Zn including spin-dependent NLD for J=6-10. An equal parity distribution is assumed.

E_x (MeV)	Total NLD (MeV^{-1})	J=6	J=7	J=8	J=9	J=10
0.000	0.00E+00	0.00E+00	0.00E+00	0.00E+00	0.00E+00	0.00E+00
0.106	8.86E-02	0.00E+00	0.00E+00	0.00E+00	0.00E+00	0.00E+00
0.212	9.59E-02	1.74E-07	1.55E-09	0.00E+00	0.00E+00	0.00E+00
0.318	1.16E-01	3.21E-06	7.59E-08	0.00E+00	0.00E+00	0.00E+00
0.424	1.44E-01	2.06E-05	8.84E-07	2.34E-08	0.00E+00	0.00E+00
0.530	1.80E-01	7.90E-05	5.13E-06	2.18E-07	6.08E-09	0.00E+00
0.637	2.27E-01	2.26E-04	1.99E-05	1.20E-06	4.97E-08	0.00E+00
0.743	2.85E-01	5.32E-04	5.98E-05	4.74E-06	2.67E-07	1.07E-08
0.849	3.58E-01	1.10E-03	1.50E-04	1.49E-05	1.07E-06	5.67E-08
0.955	4.49E-01	2.07E-03	3.32E-04	3.94E-05	3.50E-06	2.32E-07
1.061	5.60E-01	3.63E-03	6.66E-04	9.23E-05	9.74E-06	7.85E-07
1.167	6.96E-01	6.02E-03	1.24E-03	1.96E-04	2.40E-05	2.28E-06
1.273	8.63E-01	9.54E-03	2.17E-03	3.86E-04	5.38E-05	5.90E-06
1.379	1.07E+00	1.46E-02	3.62E-03	7.13E-04	1.11E-04	1.39E-05
1.485	1.31E+00	2.16E-02	5.81E-03	1.25E-03	2.16E-04	3.01E-05
1.591	1.61E+00	3.12E-02	9.01E-03	2.10E-03	3.97E-04	6.10E-05
1.697	1.97E+00	4.41E-02	1.36E-02	3.40E-03	6.97E-04	1.17E-04
1.803	2.40E+00	6.13E-02	2.00E-02	5.34E-03	1.18E-03	2.15E-04
1.909	2.93E+00	8.37E-02	2.87E-02	8.15E-03	1.92E-03	3.78E-04
2.016	3.55E+00	1.13E-01	4.06E-02	1.22E-02	3.05E-03	6.42E-04
2.122	4.30E+00	1.50E-01	5.64E-02	1.78E-02	4.71E-03	1.06E-03
2.228	5.19E+00	1.97E-01	7.72E-02	2.55E-02	7.12E-03	1.69E-03
2.334	6.25E+00	2.57E-01	1.04E-01	3.60E-02	1.05E-02	2.64E-03
2.440	7.51E+00	3.32E-01	1.40E-01	5.00E-02	1.53E-02	4.03E-03
2.546	9.01E+00	4.25E-01	1.85E-01	6.86E-02	2.19E-02	6.03E-03
2.652	1.08E+01	5.40E-01	2.42E-01	9.30E-02	3.09E-02	8.87E-03

Continued on next page

Table 5 – continued from previous page

E_x (MeV)	Total NLD (MeV^{-1})	J=6	J=7	J=8	J=9	J=10
2.758	1.29E+01	6.82E-01	3.14E-01	1.25E-01	4.29E-02	1.28E-02
2.864	1.54E+01	8.55E-01	4.05E-01	1.66E-01	5.90E-02	1.83E-02
2.970	1.83E+01	1.07E+00	5.18E-01	2.18E-01	8.01E-02	2.58E-02
3.076	2.17E+01	1.33E+00	6.58E-01	2.85E-01	1.08E-01	3.59E-02
3.182	2.58E+01	1.64E+00	8.30E-01	3.69E-01	1.44E-01	4.93E-02
3.288	3.05E+01	2.01E+00	1.04E+00	4.74E-01	1.90E-01	6.71E-02
3.395	3.61E+01	2.46E+00	1.30E+00	6.05E-01	2.49E-01	9.05E-02
3.501	4.26E+01	3.00E+00	1.62E+00	7.69E-01	3.23E-01	1.21E-01
3.607	5.02E+01	3.65E+00	2.00E+00	9.71E-01	4.18E-01	1.60E-01
3.713	5.91E+01	4.42E+00	2.47E+00	1.22E+00	5.36E-01	2.11E-01
3.819	6.95E+01	5.34E+00	3.03E+00	1.52E+00	6.84E-01	2.75E-01
3.925	8.15E+01	6.42E+00	3.70E+00	1.90E+00	8.68E-01	3.57E-01
4.031	9.56E+01	7.70E+00	4.50E+00	2.35E+00	1.10E+00	4.60E-01
4.137	1.12E+02	9.22E+00	5.47E+00	2.90E+00	1.38E+00	5.90E-01
4.243	1.31E+02	1.10E+01	6.61E+00	3.56E+00	1.72E+00	7.51E-01
4.349	1.53E+02	1.31E+01	7.98E+00	4.36E+00	2.14E+00	9.53E-01
4.455	1.78E+02	1.56E+01	9.59E+00	5.31E+00	2.66E+00	1.20E+00
4.561	2.08E+02	1.85E+01	1.15E+01	6.46E+00	3.28E+00	1.51E+00
4.668	2.42E+02	2.18E+01	1.38E+01	7.83E+00	4.04E+00	1.89E+00
4.774	2.82E+02	2.58E+01	1.64E+01	9.47E+00	4.95E+00	2.35E+00
4.880	3.27E+02	3.04E+01	1.96E+01	1.14E+01	6.05E+00	2.92E+00
4.986	3.80E+02	3.57E+01	2.33E+01	1.37E+01	7.36E+00	3.61E+00
5.092	4.41E+02	4.20E+01	2.76E+01	1.65E+01	8.94E+00	4.44E+00
5.198	5.10E+02	4.92E+01	3.27E+01	1.97E+01	1.08E+01	5.45E+00
5.304	5.91E+02	5.76E+01	3.86E+01	2.35E+01	1.31E+01	6.67E+00
5.410	6.83E+02	6.73E+01	4.55E+01	2.80E+01	1.58E+01	8.14E+00
5.516	7.90E+02	7.85E+01	5.35E+01	3.33E+01	1.89E+01	9.90E+00
5.622	9.12E+02	9.15E+01	6.29E+01	3.95E+01	2.27E+01	1.20E+01
5.728	1.05E+03	1.07E+02	7.38E+01	4.67E+01	2.72E+01	1.45E+01
5.834	1.21E+03	1.24E+02	8.65E+01	5.52E+01	3.24E+01	1.75E+01
5.940	1.40E+03	1.44E+02	1.01E+02	6.52E+01	3.86E+01	2.11E+01
6.047	1.61E+03	1.67E+02	1.18E+02	7.68E+01	4.59E+01	2.54E+01
6.153	1.85E+03	1.93E+02	1.38E+02	9.03E+01	5.45E+01	3.04E+01
6.259	2.13E+03	2.23E+02	1.61E+02	1.06E+02	6.45E+01	3.64E+01
6.365	2.45E+03	2.58E+02	1.87E+02	1.24E+02	7.63E+01	4.34E+01
6.471	2.79E+03	2.97E+02	2.16E+02	1.45E+02	9.00E+01	5.17E+01
6.577	3.11E+03	3.32E+02	2.44E+02	1.65E+02	1.03E+02	5.95E+01
6.683	3.47E+03	3.71E+02	2.74E+02	1.86E+02	1.17E+02	6.83E+01

Continued on next page

Table 5 – continued from previous page

E_x (MeV)	Total NLD (MeV^{-1})	J=6	J=7	J=8	J=9	J=10
6.789	3.85E+03	4.14E+02	3.07E+02	2.10E+02	1.33E+02	7.82E+01
6.895	4.27E+03	4.61E+02	3.43E+02	2.36E+02	1.50E+02	8.92E+01
7.001	4.72E+03	5.12E+02	3.83E+02	2.65E+02	1.70E+02	1.01E+02
7.107	5.22E+03	5.67E+02	4.27E+02	2.97E+02	1.92E+02	1.15E+02
7.213	5.75E+03	6.28E+02	4.74E+02	3.32E+02	2.15E+02	1.30E+02
7.319	6.33E+03	6.93E+02	5.26E+02	3.70E+02	2.41E+02	1.47E+02
7.426	6.96E+03	7.64E+02	5.82E+02	4.11E+02	2.70E+02	1.65E+02
7.532	7.63E+03	8.40E+02	6.43E+02	4.56E+02	3.01E+02	1.86E+02
7.638	8.36E+03	9.22E+02	7.09E+02	5.05E+02	3.35E+02	2.08E+02
7.744	9.14E+03	1.01E+03	7.79E+02	5.58E+02	3.72E+02	2.32E+02
7.850	9.97E+03	1.10E+03	8.55E+02	6.15E+02	4.13E+02	2.59E+02
7.956	1.09E+04	1.21E+03	9.37E+02	6.77E+02	4.56E+02	2.87E+02
8.062	1.18E+04	1.31E+03	1.03E+03	7.43E+02	5.03E+02	3.19E+02
8.168	1.28E+04	1.43E+03	1.12E+03	8.15E+02	5.54E+02	3.53E+02
8.274	1.39E+04	1.55E+03	1.22E+03	8.91E+02	6.09E+02	3.89E+02
8.380	1.50E+04	1.68E+03	1.33E+03	9.73E+02	6.67E+02	4.29E+02

Table 6: TALYS default γSF for ^{74}Zn.

γ-ray Energy (MeV)	f(M1) (MeV^{-3})	f(E1) (MeV^{-3})	γSF (MeV^{-3})
0.001	0.000E+00	1.108E-08	1.108E-08
0.002	1.532E-12	1.108E-08	1.108E-08
0.005	3.830E-12	1.108E-08	1.109E-08
0.010	7.660E-12	1.109E-08	1.109E-08
0.020	1.532E-11	1.110E-08	1.111E-08
0.050	3.830E-11	1.112E-08	1.116E-08
0.100	7.661E-11	1.117E-08	1.125E-08
0.200	1.533E-10	1.126E-08	1.141E-08
0.300	2.302E-10	1.135E-08	1.158E-08
0.400	3.073E-10	1.144E-08	1.175E-08
0.500	3.848E-10	1.154E-08	1.192E-08
0.600	4.628E-10	1.163E-08	1.210E-08
0.700	5.413E-10	1.173E-08	1.227E-08
0.800	6.204E-10	1.183E-08	1.245E-08
0.900	7.002E-10	1.193E-08	1.263E-08
1.000	7.809E-10	1.204E-08	1.282E-08
1.100	8.625E-10	1.214E-08	1.301E-08
1.200	9.451E-10	1.226E-08	1.320E-08
1.300	1.029E-09	1.237E-08	1.340E-08
1.400	1.114E-09	1.249E-08	1.361E-08
1.500	1.200E-09	1.262E-08	1.382E-08
1.600	1.288E-09	1.275E-08	1.403E-08
1.700	1.377E-09	1.288E-08	1.426E-08
1.800	1.468E-09	1.302E-08	1.449E-08
1.900	1.561E-09	1.317E-08	1.473E-08
2.000	1.656E-09	1.332E-08	1.498E-08
2.200	1.853E-09	1.365E-08	1.550E-08
2.400	2.059E-09	1.401E-08	1.606E-08
2.600	2.276E-09	1.440E-08	1.667E-08
2.800	2.505E-09	1.483E-08	1.733E-08
3.000	2.749E-09	1.530E-08	1.805E-08
3.200	3.008E-09	1.582E-08	1.883E-08
3.400	3.286E-09	1.639E-08	1.967E-08
3.600	3.584E-09	1.701E-08	2.059E-08
3.800	3.904E-09	1.769E-08	2.160E-08
4.000	4.251E-09	1.844E-08	2.269E-08
4.500	5.254E-09	2.064E-08	2.589E-08
5.000	6.509E-09	2.338E-08	2.989E-08
5.500	8.110E-09	2.679E-08	3.490E-08
6.000	1.019E-08	3.103E-08	4.122E-08
6.500	1.295E-08	3.628E-08	4.922E-08
7.000	1.662E-08	4.278E-08	5.940E-08
7.500	2.150E-08	5.083E-08	7.233E-08
8.000	2.776E-08	6.083E-08	8.858E-08
8.500	3.503E-08	7.326E-08	1.083E-07
9.000	4.173E-08	8.877E-08	1.305E-07
9.500	4.502E-08	1.082E-07	1.532E-07

Table 7: CoH default γSF for ^{74}Zn.

γ-ray Energy (MeV)	f(M1) (MeV^{-3})	f(E1) (MeV^{-3})	γSF (MeV^{-3})
0.05	5.419E-12	5.542E-09	5.548E-09
0.10	1.084E-11	5.531E-09	5.542E-09
0.15	1.626E-11	5.519E-09	5.535E-09
0.20	2.169E-11	5.507E-09	5.529E-09
0.25	2.713E-11	5.495E-09	5.522E-09
0.30	3.257E-11	5.483E-09	5.515E-09
0.35	3.802E-11	5.470E-09	5.508E-09
0.40	4.348E-11	5.457E-09	5.501E-09
0.45	4.896E-11	5.444E-09	5.493E-09
0.50	5.445E-11	5.431E-09	5.485E-09
0.55	5.995E-11	5.418E-09	5.478E-09
0.60	6.547E-11	5.404E-09	5.470E-09
0.65	7.102E-11	5.391E-09	5.462E-09
0.70	7.658E-11	5.377E-09	5.453E-09
0.75	8.216E-11	5.363E-09	5.445E-09
0.80	8.777E-11	5.349E-09	5.437E-09
0.85	9.341E-11	5.335E-09	5.429E-09
0.90	9.907E-11	5.321E-09	5.420E-09
0.95	1.048E-10	5.307E-09	5.412E-09
1.00	1.105E-10	5.293E-09	5.403E-09
1.05	1.162E-10	5.278E-09	5.394E-09
1.10	1.220E-10	5.264E-09	5.386E-09
1.15	1.279E-10	5.250E-09	5.377E-09
1.20	1.337E-10	5.235E-09	5.369E-09
1.25	1.396E-10	5.221E-09	5.360E-09
1.30	1.456E-10	5.206E-09	5.352E-09
1.35	1.516E-10	5.192E-09	5.343E-09
1.40	1.576E-10	5.178E-09	5.335E-09
1.45	1.637E-10	5.163E-09	5.327E-09
1.50	1.698E-10	5.149E-09	5.319E-09
1.55	1.760E-10	5.135E-09	5.311E-09
1.60	1.822E-10	5.121E-09	5.303E-09
1.65	1.885E-10	5.107E-09	5.295E-09
1.70	1.949E-10	5.093E-09	5.288E-09
1.75	2.013E-10	5.079E-09	5.280E-09
1.80	2.078E-10	5.065E-09	5.273E-09
1.85	2.143E-10	5.052E-09	5.266E-09
1.90	2.209E-10	5.038E-09	5.259E-09
1.95	2.276E-10	5.025E-09	5.252E-09
2.00	2.343E-10	5.012E-09	5.246E-09
2.05	2.412E-10	4.999E-09	5.240E-09
2.10	2.481E-10	4.986E-09	5.234E-09
2.15	2.550E-10	4.974E-09	5.229E-09
2.20	2.621E-10	4.961E-09	5.224E-09
2.25	2.693E-10	4.949E-09	5.219E-09
2.30	2.765E-10	4.938E-09	5.214E-09
2.35	2.838E-10	4.926E-09	5.210E-09
2.40	2.913E-10	4.915E-09	5.206E-09
2.45	2.988E-10	4.904E-09	5.202E-09
2.50	3.064E-10	4.893E-09	5.199E-09
2.55	3.141E-10	4.883E-09	5.197E-09
2.60	3.220E-10	4.872E-09	5.194E-09
2.65	3.299E-10	4.863E-09	5.193E-09

Continued on next page

Table 7 – continued from previous page

γ-ray Energy (MeV)	f(M1) (MeV^{-3})	f(E1) (MeV^{-3})	γSF (MeV^{-3})
2.70	3.380E-10	4.853E-09	5.191E-09
2.75	3.461E-10	4.844E-09	5.190E-09
2.80	3.544E-10	4.836E-09	5.190E-09
2.85	3.628E-10	4.827E-09	5.190E-09
2.90	3.714E-10	4.820E-09	5.191E-09
2.95	3.801E-10	4.812E-09	5.192E-09
3.00	3.889E-10	4.805E-09	5.194E-09
3.05	3.978E-10	4.799E-09	5.196E-09
3.10	4.069E-10	4.792E-09	5.199E-09
3.15	4.162E-10	4.787E-09	5.203E-09
3.20	4.256E-10	4.782E-09	5.207E-09
3.25	4.352E-10	4.777E-09	5.212E-09
3.30	4.449E-10	4.773E-09	5.218E-09
3.35	4.548E-10	4.770E-09	5.224E-09
3.40	4.649E-10	4.767E-09	5.232E-09
3.45	4.751E-10	4.764E-09	5.239E-09
3.50	4.856E-10	4.763E-09	5.248E-09
3.55	4.962E-10	4.761E-09	5.258E-09
3.60	5.070E-10	4.761E-09	5.268E-09
3.65	5.180E-10	4.761E-09	5.279E-09
3.70	5.293E-10	4.762E-09	5.291E-09
3.75	5.407E-10	4.763E-09	5.304E-09
3.80	5.524E-10	4.765E-09	5.318E-09
3.85	5.643E-10	4.768E-09	5.333E-09
3.90	5.764E-10	4.772E-09	5.348E-09
3.95	5.888E-10	4.776E-09	5.365E-09
4.00	6.014E-10	4.782E-09	5.383E-09
4.05	6.143E-10	4.788E-09	5.402E-09
4.10	6.275E-10	4.794E-09	5.422E-09
4.15	6.409E-10	4.802E-09	5.443E-09
4.20	6.546E-10	4.810E-09	5.465E-09
4.25	6.686E-10	4.820E-09	5.488E-09
4.30	6.829E-10	4.830E-09	5.513E-09
4.35	6.975E-10	4.841E-09	5.539E-09
4.40	7.125E-10	4.854E-09	5.566E-09
4.45	7.277E-10	4.867E-09	5.594E-09
4.50	7.433E-10	4.881E-09	5.624E-09
4.55	7.593E-10	4.896E-09	5.655E-09
4.60	7.756E-10	4.912E-09	5.688E-09
4.65	7.923E-10	4.930E-09	5.722E-09
4.70	8.094E-10	4.948E-09	5.757E-09
4.75	8.269E-10	4.967E-09	5.794E-09
4.80	8.448E-10	4.988E-09	5.833E-09
4.85	8.631E-10	5.010E-09	5.873E-09
4.90	8.819E-10	5.033E-09	5.915E-09
4.95	9.012E-10	5.057E-09	5.958E-09
5.00	9.209E-10	5.083E-09	6.004E-09
5.05	9.410E-10	5.110E-09	6.051E-09
5.10	9.617E-10	5.138E-09	6.100E-09
5.15	9.829E-10	5.167E-09	6.150E-09
5.20	1.005E-09	5.198E-09	6.203E-09
5.25	1.027E-09	5.231E-09	6.258E-09
5.30	1.050E-09	5.264E-09	6.314E-09
5.35	1.073E-09	5.300E-09	6.373E-09

Continued on next page

Table 7 – continued from previous page

γ-ray Energy (MeV)	f(M1) (MeV^{-3})	f(E1) (MeV^{-3})	γSF (MeV^{-3})
5.40	1.097E-09	5.336E-09	6.434E-09
5.45	1.122E-09	5.375E-09	6.497E-09
5.50	1.147E-09	5.414E-09	6.562E-09
5.55	1.174E-09	5.456E-09	6.629E-09
5.60	1.200E-09	5.499E-09	6.699E-09
5.65	1.228E-09	5.544E-09	6.771E-09
5.70	1.256E-09	5.590E-09	6.846E-09
5.75	1.285E-09	5.639E-09	6.924E-09
5.80	1.315E-09	5.689E-09	7.004E-09
5.85	1.345E-09	5.741E-09	7.086E-09
5.90	1.377E-09	5.795E-09	7.171E-09
5.95	1.409E-09	5.851E-09	7.260E-09
6.00	1.442E-09	5.908E-09	7.351E-09
6.05	1.476E-09	5.968E-09	7.445E-09
6.10	1.512E-09	6.030E-09	7.542E-09
6.15	1.548E-09	6.094E-09	7.642E-09
6.20	1.585E-09	6.160E-09	7.745E-09
6.25	1.623E-09	6.229E-09	7.852E-09
6.30	1.662E-09	6.299E-09	7.962E-09
6.35	1.703E-09	6.372E-09	8.075E-09
6.40	1.745E-09	6.448E-09	8.192E-09
6.45	1.788E-09	6.525E-09	8.313E-09
6.50	1.832E-09	6.606E-09	8.437E-09
6.55	1.877E-09	6.688E-09	8.566E-09
6.60	1.924E-09	6.774E-09	8.698E-09
6.65	1.972E-09	6.862E-09	8.834E-09
6.70	2.022E-09	6.953E-09	8.974E-09
6.75	2.073E-09	7.046E-09	9.119E-09
6.80	2.125E-09	7.142E-09	9.268E-09
6.85	2.180E-09	7.242E-09	9.421E-09

Table 8: EMPIRE default γSF for ^{74}Zn.

γ-ray Energy (MeV)	γSF (MeV^{-3})	γSF (mb/MeV)
0.106	3.223E-12	3.942E-06
0.212	1.290E-11	3.154E-05
0.318	2.903E-11	1.065E-04
0.424	5.163E-11	2.526E-04
0.530	8.073E-11	4.936E-04
0.636	1.163E-10	8.537E-04
0.743	1.585E-10	1.357E-03
0.849	2.073E-10	2.028E-03
0.955	2.626E-10	2.891E-03
1.061	3.247E-10	3.971E-03
1.167	3.935E-10	5.294E-03
1.273	4.691E-10	6.885E-03
1.379	5.516E-10	8.769E-03
1.485	6.410E-10	1.097E-02
1.591	7.374E-10	1.353E-02
1.697	8.410E-10	1.646E-02

Continued on next page

Table 8 – continued from previous page

γ-ray Energy (MeV)	γSF (MeV^{-3})	γSF (mb/MeV)
1.803	9.517E-10	1.979E-02
1.909	1.070E-09	2.355E-02
2.016	1.195E-09	2.777E-02
2.122	1.328E-09	3.249E-02
2.228	1.469E-09	3.773E-02
2.334	1.617E-09	4.352E-02
2.440	1.774E-09	4.989E-02
2.546	1.938E-09	5.689E-02
2.652	2.111E-09	6.454E-02
2.758	2.292E-09	7.288E-02
2.864	2.482E-09	8.194E-02
2.970	2.680E-09	9.177E-02
3.076	2.887E-09	1.024E-01
3.182	3.104E-09	1.139E-01
3.288	3.330E-09	1.262E-01
3.395	3.565E-09	1.395E-01
3.501	3.810E-09	1.538E-01
3.607	4.066E-09	1.690E-01
3.713	4.331E-09	1.854E-01
3.819	4.607E-09	2.028E-01
3.925	4.894E-09	2.215E-01
4.031	5.193E-09	2.413E-01
4.137	5.502E-09	2.624E-01
4.243	5.824E-09	2.849E-01
4.349	6.157E-09	3.087E-01
4.455	6.503E-09	3.340E-01
4.561	6.862E-09	3.609E-01
4.668	7.235E-09	3.893E-01
4.774	7.620E-09	4.194E-01
4.880	8.020E-09	4.512E-01
4.986	8.435E-09	4.848E-01
5.092	8.864E-09	5.204E-01
5.198	9.309E-09	5.579E-01
5.304	9.770E-09	5.974E-01
5.410	1.025E-08	6.392E-01
5.516	1.074E-08	6.832E-01
5.622	1.125E-08	7.295E-01
5.728	1.179E-08	7.783E-01
5.834	1.234E-08	8.297E-01
5.940	1.290E-08	8.838E-01
6.047	1.349E-08	9.406E-01
6.153	1.410E-08	1.000E+00
6.259	1.474E-08	1.063E+00
6.365	1.539E-08	1.129E+00
6.471	1.607E-08	1.199E+00
6.577	1.677E-08	1.272E+00
6.683	1.750E-08	1.348E+00
6.789	1.826E-08	1.429E+00
6.895	1.904E-08	1.513E+00
7.001	1.985E-08	1.602E+00
7.107	2.069E-08	1.695E+00
7.213	2.156E-08	1.793E+00
7.319	2.246E-08	1.895E+00
7.426	2.340E-08	2.003E+00

Continued on next page

Table 8 – continued from previous page

γ-ray Energy (MeV)	γSF (MeV^{-3})	γSF (mb/MeV)
7.532	2.437E-08	2.116E+00
7.638	2.538E-08	2.234E+00
7.744	2.642E-08	2.359E+00
7.850	2.751E-08	2.489E+00
7.956	2.863E-08	2.626E+00
8.062	2.980E-08	2.770E+00
8.168	3.101E-08	2.920E+00
8.274	3.227E-08	3.078E+00
8.380	3.358E-08	3.244E+00
